# Adverse Childhood Experiences and Telomere Length a Look Into the Heterogeneity of Findings—A Narrative Review

**DOI:** 10.3389/fnins.2019.00490

**Published:** 2019-05-22

**Authors:** David Bürgin, Aoife O'Donovan, Delfine d'Huart, Alain di Gallo, Anne Eckert, Jörg Fegert, Klaus Schmeck, Marc Schmid, Cyril Boonmann

**Affiliations:** ^1^Child and Adolescent Psychiatric Clinic, Psychiatric University Hospitals, University of Basel, Basel, Switzerland; ^2^Department of Psychiatry and Weill Institute for Neurosciences, University of California, San Francisco, San Francisco, CA, United States; ^3^Neurobiological Laboratory for Brain Aging and Mental Health, Transfaculty Research Platform, University of Basel, Basel, Switzerland; ^4^Child and Adolescent Psychiatry/Psychotherapy, Ulm University Medical Center, Ulm, Germany

**Keywords:** early adversity, adverse childhood experiences, stress, childhood trauma, accelerated aging, telomeres, telomere length

## Abstract

**Background:** Adverse childhood experiences (ACEs) have been associated with poor mental and somatic health. Accumulating evidence indicates that accelerated biological aging—indexed by altered telomere-related markers—may contribute to associations between ACEs and negative long-term health outcomes. Telomeres are repeated, non-coding deoxyribonucleic acid (DNA) sequences at the end of chromosomes. Telomeres shorten during repeated cell divisions over time and are being used as a marker of biological aging.

**Objectives:** The aim of the current paper is to review the literature on the relationship between ACEs and telomere length (TL), with a specific focus on how the heterogeneity of sample and ACEs characteristics lead to varying associations between ACEs and TL.

**Methods:** Multiple databases were searched for relevant English peer-reviewed articles. Thirty-eight papers were found to be eligible for inclusion in the current review.

**Results:** Overall, the studies indicated a negative association between ACEs and TL, although many papers presented mixed findings and about a quarter of eligible studies found no association. Studies with smaller sample sizes more often reported significant associations than studies with larger samples. Also, studies reporting on non-clinical and younger samples more often found associations between ACEs and TL compared to studies with clinical and older samples. Reviewing the included studies based on the “Stressor Exposure Characteristics” recently proposed by Epel et al. (2018) revealed a lack of detailed information regarding ACEs characteristics in many studies.

**Conclusion:** Overall, it is difficult to achieve firm conclusions about associations of ACEs with TL due to the heterogeneity of study and ACE characteristics and the heterogeneity in reported findings. The field would benefit from more detailed descriptions of study samples and measurement of ACEs.

## Introduction

Adverse childhood experiences (ACEs) (e.g., physical abuse, sexual abuse, emotional neglect, loss of a close family member) are a large societal problem, often with long-lasting health consequences. Previous research has shown that ACEs are highly prevalent. In the general population, more than half of people retrospectively report at least one, and more than a quarter two or more, types of ACEs (Felitti et al., 1998; Dube et al., 2001). In addition, ACEs are found to be related to poor health outcomes, including various mental health problems (e.g., depression, anxiety, post-traumatic stress disorder [PTSD], suicidal ideation), substance abuse problems, self-reported illness, obesity, and overall morbidity (Felitti et al., 1998; Widom, 1999; Dube et al., 2001, 2003; Anda et al., 2006, 2010; Widom et al., 2007; Brown et al., 2009; Green et al., 2010; Heim et al., 2010; Kessler et al., 2010; Heim and Binder, 2012; Moffitt and the Klaus-Grawe Think Tank, 2013). ACEs have also been found to be associated with increased risk for many somatic diseases, especially with diseases of aging including cancer, autoimmune, cardiovascular diseases and early mortality (Felitti et al., 1998; Brown et al., 2009; Rich-Edwards et al., 2012; Kelly-Irving et al., 2013; Tomasdottir et al., 2015). Although it is largely accepted that ACEs increase risk for poor health outcomes, mechanisms of the association are still not fully understood (Moffitt and the Klaus-Grawe Think Tank, 2013).

Following a pioneering study by Epel et al. (2004), research on the association of stress and telomere-related processes has rapidly emerged. Accelerated cell aging—indexed by altered telomere maintenance—might be one mechanism that partially explains the association between ACEs and long-term health complaints. Telomeres are repeated non-coding deoxyribonucleic acid (DNA) sequences—TTAGGG nucleotide tandem repeats – at the end of chromosomes, protecting the coded sequences (Blackburn, 1991). Telomeres shorten during cell division, caused by an incomplete replication of the chromosome ends (Blackburn, 2000, 2001). When telomeres are critically short, cells become genomically unstable and can malfunction in cell-specific ways (Blackburn, 2000). Telomeres tend to shorten with age, which makes telomere length (TL) an interesting marker of biological aging (Cawthon et al., 2003; Blackburn, 2005; Aubert and Lansdorp, 2008; Takubo et al., 2010). Interestingly, shorter telomeres are correlated with several psychiatric disorders (Lindqvist et al., 2015; Schutte and Malouff, 2015; Darrow et al., 2016; Ridout et al., 2016; Li et al., 2017a; Epel and Prather, 2018), somatic diseases (Honig et al., 2006; Willeit et al., 2010), and early mortality (Cawthon et al., 2003).

A fast-growing body of research describes the association between ACEs and TL over the life course. Various reviews in the broader context of the association between stress and TL have recently been published focusing in detail on early life stress and telomeres (Shalev, 2012; Price et al., 2013; Ridout et al., 2015), perceived stress and TL (Schutte and Malouff, 2014; Mathur et al., 2016), childhood exposure to violence and TL (Moffitt and the Klaus-Grawe Think Tank, 2013), violence and telomeres (Oliveira et al., 2016), caregiving experiences and telomeres (Blaze et al., 2015), and psychosocial factors and TL (Starkweather et al., 2014). Additionally, recent meta-analyses describe the association between early life adversity and TL (Ridout et al., 2017), childhood trauma and accelerated telomere erosion (Li et al., 2017b) and childhood psychosocial stressors and TL (Hanssen et al., 2017). Overall, these analyses reported negative associations between ACEs and TL with aggregated small effect sizes [Ridout et al. (2017) Cohen's d = −0.35; Hanssen et al. (2017) *r* = −0.082; and Li et al. (2017b) *r* = −0.05]. Epel and Prather (2018) summarized the current empirical evidence, concluding that “these meta-analyses demonstrate the robustness of the association [childhood stressors and telomere length] across published studies” (p. 5). However, all three meta-analyses reported a high between-study heterogeneity of effects, which they tried to explain in further moderator analyses. In their moderator analyses Ridout et al. (2017) showed “that differences in developmental timing of adversity exposure and comorbidities likely contributed to the heterogeneity” (p. 12), Li et al. (2017b) concluded that “the heterogeneous feature of childhood trauma may be one of the major potential sources of heterogeneity in outcomes” (p. 68), and Hanssen et al. (2017) found greater effect sizes for categorical compared to continuous measures of stressors, and for shorter durations between stressor and TL measures. Hence, a possible explanation for the observed heterogeneity in findings are attributes related to the characteristics and measurement of stressors. A deeper understanding about the different aspects of ACEs might help to explain the diversity in reported associations.

Epel and Lithgow (2014) stated that research must form a “common knowledge base and taxonomy for describing stressors and stress responses” (p. 11) to bridge the gap between basic and clinical research on aging and stress. Epel et al. (2018) further pointed out that “a large but disjointed literature shows that stress affects slow-acting biological processes in the brain and body, accelerating diseases of aging” (p. 146), but that despite this agreement one major barrier that prevents research progress is the “lack of consistency and thoroughness in stress measurement”(p. 146). This lack of a common knowledge base, consistency and thoroughness in stress measures can also be seen in the field of early life stressors and childhood adversities. Specifically, these conceptual issues lead to a large heterogeneity of reported prevalence and incidence rates of early traumatic stressors and ACEs (Heim and Binder, 2012; Moffitt and the Klaus-Grawe Think Tank, 2013). It can also be seen in the reviews and meta-analyses discussed here that use varying stress -frameworks but overall overlap to a great degree in their included studies.

In search of a common knowledge base and taxonomy, Epel et al. (2018) proposed a working model focusing on stress as “an emergent process that involves interactions between individual and environmental factors, historical and current events, allostatic states, and psychological and physiological reactivity” (p. 146). This model comprises different research perspectives on stress and introduces a more precise language for describing stress measures. Within this framework, stress consists of an exposure within in a specific context that elicits a stress-related response. Stressor exposure characteristics (SECs) are defined along different dimensions: timescale for stress measurements (acute, event-based, daily, chronic), developmental life stages of stress exposures, stress assessment windows (measurement timeframe; proximity of assessment to the stressor in years), and stressor attributes (duration, severity, controllability, life domain, target of stressor, potential of the stressor to elicit harmful response). However, it is unknown to what extend the proposed SECs can be applied to a diverse body of literature focused on ACEs and TL.

Therefore, the main aim of the current paper is to review the fast-growing body of literature on the associations between ACEs and TL order to find explanations for the heterogeneity in findings. The included sample of studies will be reviewed based on important study design characteristics and the SECs proposed by Epel et al. (2018). This will help us to better understand the complex relationship between ACEs and TL.

## Methods

To be included in the current review, studies had to report on ACEs, assessed by means of a questionnaire or interview, on TL, and on a statistical measure of association between these two. Hereinafter, ACEs are defined as the broad array of harmful, perceived traumatic stressors during a child's development before the age of 18. This includes childhood traumatic experiences, all forms of childhood maltreatment including abuse and neglect, and childhood exposure to violence, and the combination of these factors with further potentially harmful circumstances. Multiple search methods were used to avoid biased retrieval of studies (Rosenthal, 1995). First, a computerized search of relevant databases was conducted: PubMed, PsycInfo, Web of Science, and Google Scholar up to the 26th of April 2018. The following key words were used in varying combinations: “childhood adversit^*^,” “early life stress” or “childhood trauma” and “telomere length.” Second, the combination of several instruments reported in the papers to assess ACEs with “telomere length” was examined: Childhood Trauma Questionnaire [CTQ] (Bernstein et al., 1994, 2003); Childhood Trauma Interview [CTI] (Foote and Lovejoy, 1995); Adverse Childhood Experiences [ACE] Questionnaire (Felitti et al., 1998); and the Early Trauma Inventory [ETI] (Bremner et al., 2000). Third, reference lists from relevant reviews on the association between ACEs and TL (Shalev, 2012; Price et al., 2013; Ridout et al., 2015, 2017; Oliveira et al., 2016; Epel and Prather, 2018) were examined for possible additional studies. Finally, reference lists of all included papers were checked for potentially relevant additional articles. One eligible paper by Schaakxs et al. (2015) was excluded, because another paper from the same research group (Schaakxs et al., 2016) used the same sample.

A total of 38 studies were eligible for inclusion in this review. First, we collected information on the following sample characteristics: sample size, sex, age (of the sample), sample origin, study design (cross-sectional [case-control], longitudinal), sample composition, telomere assay approach, and covariates. Additionally, we collected the following ACEs characteristics: questionnaire (specific instrument [e.g., CTQ], modified specific instrument, item, score, total score), and age at adversity exposure. Further, ACEs characteristics were assessed using the proposed SECs defined by Epel et al. (2018). This included: timescale of the used stress measurement (i.e., acute, event-based, daily, chronic); developmental life stages (i.e., childhood only, adolescence only, childhood and adolescence); stress assessment window (i.e., measurement timeframe [e.g., retrospective or prospective]; proximity of assessment to the stressor in years [i.e., duration in years between exposure and assessment]); and stressor attributes (duration, severity, controllability, life domain, target of stressor, potential of the stressor to elicit harmful response). For a detailed definition of the SECs, please refer to Appendix A. “Stress typology for stress measurement” within the model proposed by Epel et al. (2018) (p.163). Moreover, main findings of the ACEs-TL association were summarized and coded (shorter, none, longer, mixed). In a second step, studies were grouped into categories: sample size (<400, >400), age (<25, 25–45, >45), sex (male, female) and population (clinical vs. non-clinical) and reviewed regarding their overall findings.

Information regarding sample characteristics, ACEs characteristics and main findings are presented in Table 1. Further information regarding main and sub-findings are presented in Table 2. Additional supplementary characteristics including the type of adversity and nature of the ACEs-TL association are provided in the supplementary materials (Supplementary Table 1). Information was extracted and coded by the first author (DB) and double checked by one of the co-authors (Dd'H). Differences in extracted information and coding were solved by further discussing these issues.

**Table 1:** Study and stressor characteristics.

	**Study characteristics**							**ACE characteristics**												**Findings**
												**Assessment window**		**Stressor attributes**						
**References**	**Sample size; sex**	**Age, mean (SD)**	**Sample origin**	**Design**	**Sample composition**	**Telomere assay**	**Covariates**	**Questionnaire**	**Age**	**Time scales**	**Life stage**	**Time frame**	**Proximity**	**Duration**	**Severity**	**Controllability**	**Life domain**	**Target**	**Potential**	**ACEs-TL**
Bersani et al., 2016	*N* = 76 0 f, 76 m	34.64 (9.17)	USA	Cross-sectional (case-control)	Combat-exposed subjects; 41 (healthy), 18 (PTSD), 17 (PTSD + MDD)	qPCR; granulocytes	Age, BMI, antidepressants and ethnicity	ETI-SR	< 18	Event-based, chronic	Whole childhood (< 18)	Retrospective period	17	X	X	X	X	X	X	shorter
Blom et al., 2015	*N* = 117 64 f, 53 m	15.8 (1.32)	USA	Cross-sectional (case-control)	*n* = 22 (MDD) *n* = 25 (controls) (CTQ only 47 participants)	qPCR; saliva	Total brain volume, MDD and matched healthy controls	CTQ	< 18	Event-based, chronic	Whole childhood (< 18)	Retrospective period	0	X		X	X	X	X	none
Boks et al., 2015	*N* = 96 0 f, 96 m	27.0 (9.1)	Netherland	Longitudinal	Clinical sample (PTSD); military combat exposure	qPCR; leukocyte	Baseline methyl. level, time interval, age, gender, alcohol consumption, cigarette smoking, military rank, length, weight, or medication	ETI-SR	< 18	Event-based, chronic	Whole childhood (< 18)	Retrospective period	9	X	X	X	X	X	X	none
Cai et al., 2015	*N* = 11,670 11,670 f	NA	China	Cross- sectional	CONVERGE study of MDD	qPCR; saliva	NA	Score	< 16	Event-based, chronic	Whole childhood (< 16)	Retrospective period	NA	X	X	X	X	X	X	shorter
Chen et al., 2014	*N* = 40 25 f, 15 m	36 (10.7)^a^	USA	Cross-sectional (case-control)	20 (MDD) 20 (controls)	qPCR; leukocyte	Age, sex- and ethnicity-matched controls	ACEs	< 18	Event-based, chronic	Whole childhood (< 18)	Retrospective period	18	X	X	X	X	X	X	mixed
Dagan et al., 2017	*N* = 78 62 f, 16 m	20.5 (1.6)	USA	Cross-sectional	Undergraduate students	qPCR; buccal cells	Age, gender, ethnicity, current income, Health-related factors, smoking history, current physical activity level, and BMI	ACEs	< 18	Event-based, chronic	Whole childhood (< 18)	Retrospective period	2	X	X	X	X	X	X	mixed
Drury et al., 2014	*N* = 80 39 f, 41 m	10.2 (2.9)	USA	Cross- sectional	High risk families	qPCR; buccal cells	Age, gender, maternal education, parental age at child conception, race	PAPA (mod.)	< 15^b^	Event-based, chronic	Children and adolescents (5 to 15)	Retrospective period	0	X	X	X	X	X	X	shorter
Glass et al., 2010	*N* = 1,874 NA	NA	UK	Cross-sectional (case-control)	CM (123/1874) Physical abuse (20/540) Sexual abuse (34/550)	southern blot; leukocyte	Age, sex, BMI, smoking	Items	NA	Event-based, chronic	Whole childhood (NA)	Retrospective period	NA	X	X	X	X	X	X	none
Guarneri-White et al., 2018	*N* = 108 60 f, 48 m	15.91 (1.65)	USA	Cross-sectional	Adolescence from larger suburban area recruited via school mailing lists and summer camps	qPCR; saliva	BMI, age, and sex	DIAS-VS CSEQ-SR	< 19^a^	Event-based, chronic	Children and adolescents (age 11–19)	Retrospective period	0	X	X	X	X	X	X	shorter
Jodczyk et al., 2014	*N* = 677 females and males	Range: 28–30	New-Zealand	Cross-sectional	Population-based; longitudinal Birth cohort; Christchurch Health and Development Study (CHDS)	qPCR; leukocyte	Sex, ethnicity and family SES at birth	Reports; PBI; CTS (mod.); total score	< 16	Event-based, chronic	Whole childhood (< 16)	Retrospective period	0–5	X	X	X	X	X	X	none
Kananen et al., 2010	*N* = 974 617 f, 357 m	49.8 (12.60)	Finland	Cross-sectional (case-control)	Epidemiological Health 2000 cohort; Anxiety disorder vs. control subjects	qPCR; leukocyte	Comorbidity, psychiatric medication, BMI, blood pressure, serum lipids, glucose, smoking, sleep, exercise	Items; total score	< 16	Event-based, chronic	Whole childhood (< 16)	Retrospective period	34	X	X	X	X	X	X	shorter
Kiecolt-Glaser et al., 2011	*N* = 132 95 f, 37 m	69.7 (10.14)	USA	cross-sectional	Caregivers vs. control subjects; 42/132 (abuse); 74/132 (adverse event)	southern blot; PBMCs	Age, sex, BMI, exercise, sleep, alcohol use, caregiving status	CTQ; total score	< 18	Event-based, chronic	Whole childhood (< 18)	Retrospective period	52	X	X	X	X	X	X	shorter
Kuffer et al., 2016	*N* = 120 57 f, 63 m	74.1 (6.1)^a^	Switzerland	Cross-sectional (case-control)	62 (child laborers, with or without PTSD), 58 (healthy controls)	qPCR; buccal cell	Age, sex, years of education, self-evaluated financial situation, depression, and mental and physical functioning	CTQ-SF	< 18	Event-based, chronic	Whole childhood (< 18)	Retrospective pe riod	56	X		X	X	X	X	longer
Levandowski et al., 2016	*N* = 176 176 f, 0 m	Subst.: 28.6 (7.3)^a^ Contr.: 68.3 (7.4)	Brazil	Cross-sectional (case-control)	Crack cocaine addiction: CRACK-ELS (*n* = 93) CRACK (*n* = 34) ELD (*n* = 49)	qPCR; blood	Age, educational level, BMI	CTQ	< 18	Event-based, chronic	Whole childhood (< 18)	Retrospective period	11	X	X	X	X	X	X	shorter
Liu et al., 2017	*N* = 894 590 f, 304 m	Range: 28–60 Median = 46	Sweden	Cross-sectional (case-control)	Longitudinal population-based cohort study (PART); 337 (recent depress-ion diagnosis), 574 (non-depressed controls)	qPCR; saliva	Age, alcohol, number of somatic diseases, sex, education, BMI, smokers, physical exercise regularly	Items; total score	< 18	Event-based, chronic	Whole childhood	Retrospective period	28	X	X	X	X	X	X	mixed
Malan-Müller et al., 2013	*N* = 128 128 f, 0 m	Range: 18–50 Mean = 29.8	South-Africa	Cross-sectional (case-control)	HIV-positive (83/128) Childhood Trauma (66/128)	qPCR; PBMCs	Age, education, BMI, trauma-subtype, traumatic life experiences, PTBS symptomatology, alcohol abuse	CTQ-SF	< 18	Event-based, chronic	Whole childhood (< 18)	Retrospective period	12	X	X	X	X	X	X	none
Mason et al., 2015	*N* = 1,135 1,135 f, 0 m	45.5 (4.1)	USA	Cross-sectional	Population-based Nurses' Health Study II (NHSII)	qPCR; leukocyte	Age, own and parental education, parental occupation, parental morbidity before age 60	CTS; SES	< 18	Event-based, chronic	Whole childhood (< 18)	Retrospective period	28	X		X	X	X	X	mixed
McFarland et al., 2017	*N* = 1,108 594 f, 514 m	45.6 (11.6)	USA	Cross-sectional	Nashville Stress and Health Study (NSHS)	qPCR; leukocyte	Age, gender, depressive symptoms	LHC	< 18	Event-based, chronic	Whole childhood (< 18)	Retrospective period	28	X	X	X	X	X	X	shorter
Mitchell et al., 2018	*N* = 81 81 f, 0 m	25.48 (4.27)	USA	Cross-sectional	Pregnant women from the Ohio State University Wexner Medical Center (OSUWMC)	qPCR; PBMCs	Age, race, current household, income, education level, marital status, BMI, exercise, smoking and depressive symptoms	CTQ	< 18	Event-based, chronic	Whole childhood (< 18)	Retrospective period	7	X	X	X	X	X	X	none
Mitchell et al., 2014	*N* = 40 0 f, 40 m	9	USA; African American Boys	Cross-sectional (case-control)	Fragile Families and Child Wellbeing Study (FFCWS)	qPCR; saliva	Mother's age at birth, BMI	CTS (mod.)	< 10	Event-based, chronic	Childhood (< 10)	Retrospective period	0	X	X	X	X	X	X	shorter
O'Donovan et al., 2011	*N* = 88 45 f, 43 m	30.55 (7.44)	USA	Cross-sectional (case-control)	43 adults with chronic PTSD (*n* = 18 with multiple childhood trauma) and 47 controls	qPCR; leukocyte	Age, sex, BMI, smoking, education	LSC (mod.)	< 15	Event-based, chronic	Whole childhood (< 15)	Retrospective period	16	X	X	X	X	X	X	shorter
Oliveira et al., 2017	*N* = 83 83 f, 0 m	Range: 65–74	Brazil	Cross-sectional	42 women with less than secondary education and 41 with secondary or more education	qPCR; leukocyte	Age, parental abuse of alcohol	Items	< 16	Event-based, chronic	Whole childhood (< 16)	Retrospective period	54	X	X	X	X	X	X	longer
Osler et al., 2016	*N* = 324 0 f, 324 m	56	Denmark	Cross-sectional	Copenhagen metropolitan birth- cohort	qPCR; leukocyte	Chronic diseases, and lifestyle, BMI, body weight and height	Items; total score	< 18	Event-based, chronic	Whole childhood (< 18)	Retrospective period	38	X	X	X	X	X	X	shorter
Puterman et al., 2016	*N* = 4598 2,724 f, 1874 m	Median: 70 Range: 50–90	USA	Cross-sectional	US Health and Retirement Study: longitudinal, nationally representative sample of >26,000 US residents	qPCR; saliva (cell type not specified)	Age, ethnicity, sex, education, current partnership status, BMI, smoking, medical conditions, high blood pressure, diabetes, cancer, lung disease, heart disease, stroke, psychiatric problems, and arthritis	Items; total score	< 18	Event-based, chronic	Whole childhood (< 18)	Retrospective period	52	X	X	X	X	X	X	shorter
Revesz et al., 2016	*N* = 2,936 1,950 f, 986 m	Baseline: 41.8 (13.1) Follow-up: 48.5 (13.0)	Netherlands	Longitudinal (6-year follow-up)	Population-based	qPCR; leukocyte	Sex, age, smoking, triglycerides, BP	CTI	< 16	Event-based, chronic	Whole childhood (< 16)	Retrospective period	26	X	X	X	X	X	X	mixed
Riley et al., 2018	*N* = 66 27 f, 39 m	40.9 (9.8)		Cross-sectional (case-control)	48 adults with DSM-5 schizophrenia and 18 healthy controls	qPCR; lymphocytes	Age	ETI	< 18	Event-based, chronic	Whole childhood (< 18)	Retrospective period	23	X		X	X	X	X	mixed
Robles et al., 2016	*N* = 39 28 = f, 19 m	11.3 (1.5) Range = 8–13	USA	Cross-sectional	Population-based	qPCR; leukocyte	Age, gender, BMI, parent educational status and leukocyte composition	Score; total score	[8; 13]	Daily, chronic	Childhood (age 8–13)	Daily ratings for 56 days	0	X	X	X	X	X	X	shorter
Savolainen et al., 2014	*N* = 1,486 817 f, 674 m	61.5 (2.9)	Finland	Cross-sectional	Population-based; Helsinki Birth Cohort Study	qPCR; leukocyte	Age, sex, stock of DNA, mental disorder, depression, education, alcohol, father's education and mother's age at delivery, coronary heart disease and stroke, BMI, diabetes II	National Archive Finland; TEC	Life time (median 10)	Event-based, chronic	Whole childhood + early childh., childhood and adolescence	Retrospective period, archive information	0 + 42	X	X	X	X	X	X	mixed
Schaakxs et al., 2016	*N* = 496 323 f, 173 m	70.6 (7.4)	Netherlands	Cross-sectional	Netherlands Study of Depression in Older Persons (NESDO); 44.2% childhood abuse at least once, 23.4% one or more events	qPCR; leukocyte	Sex, age, chronic disease	Score	< 16	Event-based, chronic	Whole childhood (< 16)	Retrospective period	55	X	X	X	X	X	X	mixed
Shalev et al., 2013	*N* = 236 116 f, 120 m	Baseline: 5 Follow-up: 10	UK	Longitudinal	Population-based; Environmental-Risk (E-Risk) Study, subset of epidemiological sample; 108/236 (Exp./N)	qPCR; buccal cells	Age, sex, BMI, SES	CTS; clinical interview; items; total score	[5; 10]	Event-based, chronic	Childhood (age 5–10)	Prospective, longitudinal + retrospective period	0	X	X	X	X	X	X	shorter
Shalev et al., 2014	*N* = 1037 498 f, 539 m	Baseline: 26 Follow- up: 38	New- Zealand	Longitudinal	Population- based; Dunedin Multidisciplinary Health and Development Study, birth cohort; *n* = 234: int. dis. *n* = 524: no int. dis.	qPCR; leukocyte	CM, tobacco smoking, substance dependence, psychiatric medication use	Total Score	[3; 11]	Event-based, chronic	Childhood (age 3–11)	Prospective, longitudinal + retrospective periods	0–15	X	X	X	X	X	X	none
Surtees et al., 2011	*N* = 4,441 4,441 f, 0 m	Median: 62 Range: 41–80	UK	Cross-sectional	European Prospective Investigation into Cancer (EPIC)-Norfolk Population Study	qPCR; lymphocyte	Age, physical health score, self- reported health, social class, obesity, smoking, preexisting disease	HLEQ	< 17	Event-based, chronic	Whole childhood (< 17)	Retrospective period	45	X	X	X	X	X	X	shorter
Tyrka et al., 2010	*N* = 31 22 f, 9 m	26.9 (10.1)	USA	Cross-sectional (case-control)	No history of CM (*n* = 21) or a history of moderate or severe CM (*n* = 10)	qPCR; leukocyte	Age, sex, oral contraceptives, smoking, BMI, race, education, SES, perceived stress	CTQ	< 18	Event-based, chronic	Whole childhood (< 18)	Retrospective period	9	X	X	X	X	X	X	shorter
Tyrka et al., 2016	*N* = 289 177 f, 113 m,	31.0 (10.7)	USA	Cross-sectional (case-control)	No disorder/no adversity (*n* = 113) No disorder/ adversity (*n* = 66) Disorder/no adversity (*n* = 39) Disorder/adversity (*n* = 72)	qPCR; leukocyte	Race, smoking, oral contraceptive, psychiatric disorder	CTQ	< 18	Event-based, chronic	Whole childhood (< 18)	Retrospective period	13	X	X	X	X	X	X	shorter
van Ockenburg et al., 2015	*N* = 1,094 588 f, 506 m	53.1 (11.4)	Netherlands	Longitudinal (prospective)	Population-based	qPCR; leukocyte	Sex, age, comorbidity, BMI, smoking, exercise, education	LTE (mod.)	< 12	Event-based, chronic	Childhood (< 12)	Retrospective period	41	X	X	X	X	X	X	none
Verhoeven et al., 2015	*N* = 2,936 1,950 f, 986 m	41.8 (13.1)	Netherlands	Cross-sectional	Longitudinal cohort study examining the course and consequences of depressive and anxiety disorders; 57% current depression	qPCR; leukocyte	Sex, age, comorbidity, depression, BMI, smoking, alcohol use, exercise, education	CTI; CTQ; score	< 16	Event-based, chronic	Whole childhood (< 16)	Retrospective period	26	X	X	X	X	X	X	none
Vincent et al., 2017	*N* = 180 103 f, 77 m	50 (15.65)	UK	CROSS- sectional	80 depressed subjects and 100 control subjects	qPCR; blood (cell type not specified)	Age, sex	CTQ	< 18	Event-based, chronic	Whole childhood (< 18)	Retrospective period	32	X	X	X	X	X	X	mixed
Zalli et al., 2014	*N* = 333 167 f, 166 m	63.2 (5.5)^a^	UK	Cross-sectional	Heart Scan Study, a subsample of the Whitehall II epidemiological cohort	qPCR; PBMC	Age, SES and BMI	Item	< 16	Event-based, chronic	Whole childhood (< 16)	Retrospective period	47	X	X	X	X	X	X	shorter

*ACEs, adverse childhood experiences; BMI, body mass index; BP, blood pressure; BTL, buccal cell telomere length; CA, childhood adversity; CM, childhood maltreatment; CPA, child physical abuse; CSEQ, children's social experiences questionnaire; CTI, childhood trauma interview; CTQ, childhood trauma questionnaire; CTS, conflict tactics scale; DIAS-VS, direct and indirect aggression scale-victim version; DNA, deoxyribonucleic acid; DNAm age, DNA methylation age; ELD, elderly people; ELS, early life stress; ETI, early trauma inventory; Exp., exposed; f, female; HIV, human immunodeficiency virus; HC, healthy controls; HLEQ, health and life experiences questionnaire; int.dis., internalizing disorders; LHC, life history calendar; LSC, life stressor checklist; LTE, list of threatening events; LTL, leukocyte telomere length; m, male; MDD, major depressive disorder; mod., modified; N, number of participants; NA, not available; NLE, adulthood negative life events; PBI, parental bonding instrument; PAPA, preschool age psychiatric assessment; PBMC, peripheral blood mononuclear cells; PTSD, posttraumatic stress disorder; qPCR, quantitative polymerase chain reaction; SES, socioeconomic status; SF, short form; SR, self-report; SLE, stressful life events in early life; TA, Telomerase Activity; TEC, traumatic experiences checklist; TL, Telomere length; UK, United Kingdom; USA, United States of America*.

a *weighted mean ages*.

b *Range up till the age of participants (oldest participant 15 years)*.

**Table 2:** Overview results.

	**Shorter**	**None**	**Longer**	**Mixed**
**Total association**				
ACEs and TL (*N* = 38)	18	9	2	9
ACEs and ΔTL (*N* = 5)	2	3	–	–
**Sub-findings**				
**Sample size (*****N*** **=** **38)**				
< 400 (*N* = 23)	13	4	2	4
>400 (*N* = 15)	5	5	–	5
**Sex (*****N*** **=** **37)**				
Only male (*N* = 3)	2	1	–	–
Only female (*N* = 7)	3	2	1	1
Both (*N* = 27)	13	5	1	8
**Age (*****N*** **=** **36)**				
< 25 (*N* = 7)	5	1	–	1
25–45 (*N* = 13)	5	6	–	2
>45 (*N* = 16)	7	1	2	6
**Sample Composition (*****N*** **=** **38)**				
Clinical (*N* = 16)	6	5	1	4
Non-clinical (*N* = 22)	12	4	1	5

*ΔTL, telomere attrition or within subject TL change*.

## Results

### Study Characteristics

A total of 38 studies were included in this review based on the criteria of eligibility defined in the method section (for an overview see Table 1). Sample sizes of included studies ranged from 31 (Tyrka et al., 2010) to 11,670 (Cai et al., 2015). Most studies (*N* = 27) reported on TL in both males and females, seven studies examined only females (Surtees et al., 2011; Malan-Müller et al., 2013; Cai et al., 2015; Mason et al., 2015; Levandowski et al., 2016; Oliveira et al., 2017; Mitchell et al., 2018), and three studies examined only males (Mitchell et al., 2014; Boks et al., 2015; Bersani et al., 2016; Osler et al., 2016). The included studies covered a wide age range of study participants at TL assessment from 5 years (Shalev et al., 2013; Drury et al., 2014) to 93 years of age (Schaakxs et al., 2016). Almost all of the included studies (*N* = 32) are of North-American or European origin, except for six studies that were conducted in Brazil (Levandowski et al., 2016; Oliveira et al., 2017), China (Cai et al., 2015), South-Africa (Malan-Müller et al., 2013), and New Zealand (Jodczyk et al., 2014; Shalev et al., 2014).

Reviewing the design of the studies, all studies, as defined within the inclusion criteria, had to report on TL at a minimum of one time point, and thus were able to associate ACEs and TL cross-sectionally. Of the 38 studies, 14 used a cross-sectional (case-control) approach to investigate differences in TL between groups (e.g., abused vs. non abused) (Glass et al., 2010; Kananen et al., 2010; Tyrka et al., 2010, 2016; O'Donovan et al., 2011; Malan-Müller et al., 2013; Chen et al., 2014; Mitchell et al., 2014; Blom et al., 2015; Bersani et al., 2016; Kuffer et al., 2016; Levandowski et al., 2016; Liu et al., 2017; Riley et al., 2018). Five studies measured TL at more than one time point and were therefore able to examine TL longitudinally (Shalev et al., 2013, 2014; Boks et al., 2015; van Ockenburg et al., 2015; Revesz et al., 2016). The type of samples and the sample composition of the included papers varied widely. Some studies examined general population samples, such as birth cohorts (Jodczyk et al., 2014; van Ockenburg et al., 2015; Osler et al., 2016), whereas others had a focus on specific clinical populations, such as on depressed patients (Chen et al., 2014; Blom et al., 2015; Cai et al., 2015; Liu et al., 2017; Vincent et al., 2017), patients with anxiety disorders (Kananen et al., 2010), patients with post-traumatic stress disorder (PTSD) (O'Donovan et al., 2011; Boks et al., 2015; Kuffer et al., 2016), or patients with substance use disorders (Levandowski et al., 2016).

Because there are different ways to measure telomere length (Montpetit et al., 2014), information on the telomere assay method was collected. In our sample of eligible papers, almost all studies (*N* = 36) investigated TL using a quantitative polymerase chain reaction (qPCR). Only two papers used a southern blot analysis as TL assay method (Glass et al., 2010; Kiecolt-Glaser et al., 2011). TL was examined in different cell types: six papers reported that DNA was extracted from saliva samples (Kiecolt-Glaser et al., 2011; Mitchell et al., 2014; Blom et al., 2015; Cai et al., 2015; Puterman et al., 2016; Liu et al., 2017; Guarneri-White et al., 2018), and four studies used epithelial buccal cells (Shalev et al., 2013; Drury et al., 2014; Kuffer et al., 2016; Dagan et al., 2017). The other studies (*N* = 28) extracted DNA from peripheral blood samples. Most of these studies assayed leukocyte DNA for TL (*N* = 22), four studies extracted DNA from peripheral blood monocular cells (PBMCs) (Kiecolt-Glaser et al., 2011; Malan-Müller et al., 2013; Zalli et al., 2014; Mitchell et al., 2018), and two studies extracted DNA from lymphocytes (Surtees et al., 2011; Riley et al., 2018). Although a wide variety of covariates were included across the studies, almost all studies controlled for age, sex, body mass index (BMI) and smoking.

### ACEs Characteristics

Assessments of ACEs varied substantially across studies (see Table 1). Studies examined various age ranges: 18 studies included ACEs before the age of 18 (Tyrka et al., 2010, 2016; Kiecolt-Glaser et al., 2011; Malan-Müller et al., 2013; Chen et al., 2014; Boks et al., 2015; Mason et al., 2015; Bersani et al., 2016; Kuffer et al., 2016; Levandowski et al., 2016; Osler et al., 2016; Puterman et al., 2016; Dagan et al., 2017; Liu et al., 2017; McFarland et al., 2017; Vincent et al., 2017; Mitchell et al., 2018; Riley et al., 2018), one study reported on ACEs before the age of 17 (Surtees et al., 2011), eight studies investigated ACEs before the age of 16 (Kananen et al., 2010; Jodczyk et al., 2014; Zalli et al., 2014; Cai et al., 2015; Verhoeven et al., 2015; Revesz et al., 2016; Schaakxs et al., 2016; Oliveira et al., 2017), one study before the age of 15 (O'Donovan et al., 2011) and two studies before the age of 12 (Shalev et al., 2014; van Ockenburg et al., 2015). Additionally, six studies assessed ACEs up till the time of assessment (Shalev et al., 2013; Drury et al., 2014; Mitchell et al., 2014; Blom et al., 2015; Robles et al., 2016; Guarneri-White et al., 2018). Furthermore, the eligible papers used different ACEs assessments. About half of the studies (*N* = 20) used standardized validated questionnaires or interviews to assess adversities. The most commonly used questionnaire was the retrospective, self-report CTQ (Bernstein et al., 1994) that was used in 10 studies (Tyrka et al., 2010, 2016; Kiecolt-Glaser et al., 2011; Malan-Müller et al., 2013; Blom et al., 2015; Verhoeven et al., 2015; Kuffer et al., 2016; Levandowski et al., 2016; Vincent et al., 2017; Mitchell et al., 2018). The other studies (*N* = 18) used modified versions of other questionnaires or interviews or used novel items to create adversity scores (see Table 1, column assessment; Supplementary Material, column type of adversity).

With the SECs in mind, it was shown that almost all studies (*N* = 37) either had an event-based, or event-based/chronic stress measurement timescale. The only exception was Robles et al. (2016), who based their adversity score on current ratings of daily emotions to family conflict. Regarding the developmental life stage, all papers reported on ACEs before the age of 18. Most studies did not differentiate between childhood and adolescence. However, certain studies only included ACEs in childhood or did differentiate between childhood and adolescence (Shalev et al., 2013; Drury et al., 2014; Mitchell et al., 2014; Blom et al., 2015; Robles et al., 2016; Guarneri-White et al., 2018). Some studies used smaller age ranges (Shalev et al., 2014) or built subcategories of their larger ranges (Savolainen et al., 2014; van Ockenburg et al., 2015). Looking at the stress assessment window—in particular the measurement timeframe of ACEs assessments—most studies (*N* = 34) assessed ACEs retrospectively. Some studies used combined retrospective and prospective assessments (Shalev et al., 2013, 2014), a combination of retrospective self-reports and archive information (Savolainen et al., 2014), or an adversity score based on daily ratings (Robles et al., 2016). In terms of the time between the ACEs exposure and the age at ACEs assessment, the duration varied between 0 and 56 years. Aggregating all duration measures across studies, the mean time between the end of the ACEs measure and age at ACEs assessment was approximately 23 years.

Regarding the six reviewed stressor attributes, almost no information is included and specified in the included sample of studies. First, only one study reported on the duration of ACEs (the duration of being separated from their parents) (Savolainen et al., 2014). Second, four studies reported on the severity of ACEs on a continuous scale (Blom et al., 2015; Mason et al., 2015; Kuffer et al., 2016; Riley et al., 2018). Most studies (*N* = 34), however, did not report on the severity of the stressor on a continuous measure. Instead, they reported exposure categories, defined by using self-developed items or certain cut-off scores on continuous measures. Third, none of the studies explicitly measured controllability on a continuous scale. Fourth, looking at specific life-domains, no study reported on ACEs from a specific life-domain. However, many ACEs in childhood are of interpersonal and interpersonal-intimate nature, resulting from multiple life domains, mainly family, peers and school. Fifth, no study explicitly reported on the attribute “target of the stressor,” though, most studies assessed ACEs that targeted participants themselves, or close others. Last, focusing on the attribute “potential of the stressor to elicit potential harmful responses,” none of the study described in detail the qualities inherent to the adversities that were measured.

Overall, the eligible studies reported on stressors from a broad range of potentially harmful experiences. However, a lot of information is unknown, missing or not specified. Therefore, more research using a common language and taxonomy to describe certain characteristics of stressors—in particular with regard to ACEs—is needed.

### Main Findings: ACEs and TL

In total, 18 paper reported a negative association between ACEs and TL or higher odds for shortened TL among individuals reporting exposure to ACEs compared to those who were less or non-exposed (Kananen et al., 2010; Tyrka et al., 2010, 2016; Kiecolt-Glaser et al., 2011; O'Donovan et al., 2011; Surtees et al., 2011; Shalev et al., 2013; Drury et al., 2014; Mitchell et al., 2014; Zalli et al., 2014; Cai et al., 2015; Bersani et al., 2016; Levandowski et al., 2016; Osler et al., 2016; Puterman et al., 2016; Robles et al., 2016; McFarland et al., 2017; Guarneri-White et al., 2018). Additionally, nine papers showed no association between ACEs and TL (Glass et al., 2010; Malan-Müller et al., 2013; Jodczyk et al., 2014; Shalev et al., 2014; Blom et al., 2015; Boks et al., 2015; van Ockenburg et al., 2015; Verhoeven et al., 2015; Mitchell et al., 2018). Furthermore, two studies even reported a trend toward longer telomeres among individuals reporting more ACEs (Kuffer et al., 2016; Oliveira et al., 2017). Finally, nine papers reported mixed findings, with studies reporting some associations within their data, but no conclusive association within their total sample (Chen et al., 2014; Savolainen et al., 2014; Mason et al., 2015; Revesz et al., 2016; Schaakxs et al., 2016; Dagan et al., 2017; Liu et al., 2017; Vincent et al., 2017; Riley et al., 2018).

Beyond that, five studies have examined TL at more than one time point (Shalev et al., 2013, 2014; Boks et al., 2015; van Ockenburg et al., 2015; Revesz et al., 2016). Hence, these studies were able to assess telomere attrition, which is the change in telomere length within a subject. Two of these studies showed ACEs to be associated with TL change (Shalev et al., 2013; Revesz et al., 2016), whereas three papers reported no association between ACEs and TL change (Shalev et al., 2014; Boks et al., 2015; van Ockenburg et al., 2015).

### Possible Moderators

To attempt to explain the variety in findings, comparisons were made based on sample size, age, sample composition, and sex of study samples. First, focusing on the study characteristics, the results of studies with more than 400 participants (*N* = 15) seemed to be less conclusive than studies with < 400 participants (*N* = 23). Of these studies with larger samples, five papers reported a cross-sectional association between early adversity and TL (Kananen et al., 2010; Surtees et al., 2011; Cai et al., 2015; Puterman et al., 2016; McFarland et al., 2017), five studies reported mixed results (Savolainen et al., 2014; Mason et al., 2015; Revesz et al., 2016; Schaakxs et al., 2016; Liu et al., 2017), and five reported no associations (Glass et al., 2010; Jodczyk et al., 2014; Shalev et al., 2014; van Ockenburg et al., 2015; Verhoeven et al., 2015). Second, subdividing the age of study samples indicated that studies investigating TL during childhood, adolescence or emerging adulthood (*N* = 7) more often find associations of ACEs and shorter TL (Shalev et al., 2013; Drury et al., 2014; Mitchell et al., 2014; Blom et al., 2015; Robles et al., 2016; Dagan et al., 2017; Guarneri-White et al., 2018). Findings in older samples are more inconclusive. Third, considering the sample composition, comparing clinical (with mental disorders) (*N* = 16) and non-clinical samples (without mental disorders) (*N* = 22) indicated that studies in non-clinical samples more often find negative associations between ACEs and TL than do studies in clinical populations. Fourth, with regard to the sex of participants, there were no observable differences in reported results.

## Discussion

The aim of the current review was to review the literature on the associations between ACEs and TL in an attempt to highlight how heterogeneity in sample and stressor characteristics contributes to findings. Overall, the sample of studies we reviewed indicates a negative association between ACEs and TL, although many papers presented mixed findings and a quarter of eligible studies found no relationship between ACEs and TL. These findings are consistent with recently published meta-analyses investigating the association between early adversity, childhood trauma and childhood psychosocial stressors and TL. All three studies showed significant small negative associations with TL (Hanssen et al., 2017; Li et al., 2017b; Ridout et al., 2017). These meta-analyses further reported high between-study heterogeneity of effects. Considering possible moderators within our sample of studies indicates that results of larger samples seem to be less conclusive than results of smaller samples. In addition, studies investigating participants younger than 25 more often find ACEs to be negatively associated with TL compared to older samples. Furthermore, results from studies of non-clinical samples more often report negative associations between ACEs and TL than do studies of clinical samples. Using the SECs proposed by Epel et al. (2018) to examine characteristics of the included ACEs shows a lack of detailed information on SECs in many studies. At least four findings (sample size, age, psychopathology, and ACEs characteristics) need to be discussed in more detail to find explanations for the heterogeneity and inconclusiveness of reported findings.

First, with regard to sample size, we observed that findings of larger samples are less conclusive compared to findings of smaller samples. This might be explained by the fact that larger samples can control for more additional variables and potential confounds. These additional factors might moderate, mediate, conceal or suppress the direct, independent impact of ACEs, as many of these variables in larger models are inter-correlated (e.g., adversities, mental health problems, negative life-styles, and smoking status).

Second, we observed that studies with younger participants more often find negative associations than studies with older participants. This is in line with Ridout et al. (2017) who reported in their moderator analyses that the smaller the duration between ACEs exposure and age at TL assessment, the larger the magnitude of effect sizes. They explained this finding by pointing to the fact that studies of children assume no smoking amongst participants, and that adversities early in childhood tend to be associated with larger effects (Ridout et al., 2017). Similar results were found by Hanssen et al. (2017). Another potential explanation, according to the healthy survivor effect, might be that participants within older samples drop out due to morbidity or early mortality, which is in turn associated with shorter telomeres (Mather et al., 2011; Kuffer et al., 2016; Schaakxs et al., 2016; Oliveira et al., 2017). Moreover, Schaakxs et al. (2016) argued that “a possible explanation for these null findings in older adults may be that older adults have been exposed to numerous competing causes for shortened TL, such as somatic diseases or an unhealthy lifestyle over the life span. These other TL-damaging factors may suppress the independent impact of psychosocial stressors.” (p. 441).

Third, the sample composition of included studies varied strongly. Some of the studies focused on specific clinical populations and the impact of psychiatric disorders on TL. These studies included ACEs in their models as control variables. In contrast, other studies focused on the impact of ACEs on TL controlling for psychiatric conditions. We observed that studies with non-clinical populations more often report negative associations between ACEs and TL. This is in line with Ridout et al. (2017), who found effect sizes of smaller magnitude regarding the association of ACEs and TL in their moderator analyses, when looking at studies that included subjects with mental disorders. Epel and Prather (2018) recently proposed a triad model of stress exposures, psychopathology and telomere biology combining the meta-analytic evidence between the associations of stress and telomeres, stress and psychopathology, and psychopathology and telomeres. Having this triad in mind, when approaching TL from a psychopathological perspective, studies have to acknowledge that “expression of psychopathology may be strongly influenced by exposure to maltreatment” (Teicher and Samson, 2013, p. 1,114). This distinctive phenotypical expression of a psychiatric disorder (with vs. without maltreatment) might reveal distinct subtypes of disorders that are important to account for when determining the biological bases of these mental disorders (Teicher and Samson, 2013; Teicher et al., 2016). Moreover, possible direct associations of ACEs on TL might be mediated by the later development of mental disorders. Assuming that early adversities often precede psychopathology, psychiatric disorders might mediate the association of ACEs and TL. Hence, research on TL should acknowledge both perspectives: distinct subtypes of psychiatric disorders (with vs. without maltreatment) within clinical samples and the potential mediating effect of psychopathology in non-clinical samples.

Fourth, the current study further examined ACEs using the SECs recently proposed by Epel et al. (2018). Results showed an overall lack of details and lots of missing information. This makes it indeed very difficult to understand the adverse nature of these experiences with important characteristics and attributes not being measured or articulated. Differentiating between event-based and chronic exposures, the target of the exposure, and the duration, for instance, is very important in the context of trauma research as many childhood adversities are interpersonal and traumatic in nature (e.g., abuse and neglect, interpersonal loss, interpersonal conflict, interpersonal violence) and are targeted at either participants themselves or at close others (e.g., siblings or family members) (Widom et al., 2008; Moffitt and the Klaus-Grawe Think Tank, 2013). Chronic-occurring interpersonal events are often followed by a broad range of trauma-associated psychopathologies that are not captured within the classical framework of PTSD (Cook et al., 2005). These harmful responses can lead to diverse behavioral and emotional alterations, often referred to as complex trauma symptoms, as for example affective dysregulation, attentional and behavioral problems, self and relational deregulation (Briere et al., 2008; Greeson et al., 2011; Schmid et al., 2013). For this reason many experts emphasized the need for a more developmentally sensitive diagnostic system that takes account of the heterogeneity of psychopathology following early trauma (Cloitre et al., 2009; van der Kolk et al., 2009; D'Andrea et al., 2012; Schmid et al., 2013). This led to the inclusion of complex trauma symptoms within the PTSD section in the Diagnostic and Statistical Manual of Mental Disorders—Fifth Edition (DSM-5) and the inclusion of a complex PTSD disorder in the International Classification of Diseases 11th Revision (ICD-11). These complex trauma symptoms contain symptoms of affect dysregulation, negative self-concepts and interpersonal problems that are related to the traumatic exposure (Cloitre et al., 2013). Overall, the adversities included are all of a stressful, adverse, and traumatic nature. Most of these stressors have the potential to elicit harmful emotional responses (e.g., social threat, loss of control, shame) and behavioral alterations (e.g., role-change, impulsivity), but detailed and differentiating information is missing.

### Limitations

The current review needs to be seen in light of some limitations. First, this review is not a systematic review as defined by PRISMA or Cochrane guidelines. The narrative approach, however, allowed us to discuss the complexity of exposure characteristics in an overall heterogenous sample of studies and adds to recently published systematic meta-analyses. Second, most studies assessed ACEs retrospectively with self-reported questionnaires, sometimes with several decades between adversity and assessment of adversity, which leads to recall biases. Hardt and Rutter (2004) extensively discussed biases of retrospective self-reports and concluded that they easily lead to an underreporting of events and that the validity of details assessed retrospectively might be low, but false-positive reports are rare. In contrast, a recently published meta-analysis reported only weak associations between prospective and retrospective measures of adversity concluding that these measures identify different groups of individuals (Baldwin et al., 2019). This should be taken into account in future studies. Third, this review focused on the ACEs part of the ACEs-TL association. Besides that, methodological issues with regard to the TL measurement approach are also of high interest and might explain some of the heterogeneity in findings. These issues are extensively reviewed and discussed elsewhere and beyond the scope of this review (Montpetit et al., 2014; Lai et al., 2018). Fourth, publication bias is likely to occur because we only included papers that were published in peer-reviewed journals. Last and most important, as described in the method section, studies were included that measured ACEs before the age of 18 by means of a questionnaire or an interview. Studies reporting on early adversities solely based on high-risk status, on low socio-economic status (SES), on neglectful, non-supportive parenting styles, on maternal depression, and on maternal stressors during pregnancy, were not included due to their lack of direct measurement of adverse experiences. Being at risk for ACEs is highly correlated with incidence of ACEs but not all at-risk individuals are exposed. This approach was used because the focus of this review was on the harmful long-term consequences of experiencing ACEs. Still, as a substantial overlap between different operationalization's of stressors exist, it is therefore very difficult to draw clear boundaries.

### Implications

Future research might benefit from a differentiated look into ACEs, articulating multiple domains of stressors such as in the SECs (Epel et al., 2018). This will help to improve our understanding of the adverse nature of these exposures and uncover different exposure-related emotional and behavioral responses that mediate the association between ACEs and long-term health outcomes. This might help to further our understanding of the complex associations of stress and TL, beyond what can be explained by simply summing potentially harmful incidents in childhood. In addition, resilience factors that protect children and adolescents from sustained physiological consequences need further investigation.

## Conclusion

Overall, the included sample of studies indicates a negative association between ACEs and TL, but the diversity in sample and stressor characteristics makes it difficult to achieve a final and confident conclusion. From a developmental perspective, a more comprehensive evaluation of adversities using a common language and dimensional approaches to SECs might help to improve understanding of the complex associations between (early) stressors and health outcomes. Individuals are exposed to numerous competing and interacting exposures that might shorten TL over the life course. A focus on developmental trajectories combining early adversities, psychopathology and protective factors might help to develop enhanced approaches to reduce the stress-related health burden of our societies.

## Author Contributions

DB, CB, MS, and KS contributed to the conception of the paper. DB extracted all study and stressor characteristics and wrote the first draft of the manuscript. AOD, CB and DdH wrote sections of the manuscript and edited the paper. AOD, AE, JF, AdG, MS, KS, and CB critically revised the paper. All authors contributed to manuscript revision, read and approved the submitted version.

### Conflict of Interest Statement

The authors declare that the research was conducted in the absence of any commercial or financial relationships that could be construed as a potential conflict of interest.

## References

[B1] Anda R. F. Butchart A. Felitti V. J. Brown D. W. (2010). Building a framework for global surveillance of the public health implications of adverse childhood experiences. Am. J. Prev. Med. 39, 93–98. 10.1016/j.amepre.2010.03.015 20547282

[B2] Anda R. F. Felitti V. J. Bremner J. D. Walker J. D. Whitfield C. Perry B. D.. (2006). The enduring effects of abuse and related adverse experiences in childhood. A convergence of evidence from neurobiology and epidemiology. Eur. Arch. Psychiatry Clin. Neurosci. 256, 174–186. 10.1007/s00406-005-0624-4 16311898 PMC3232061

[B3] Aubert G. Lansdorp P. M. (2008). Telomeres and aging. Physiol. Rev. 88, 557–579. 10.1152/physrev.00026.2007 18391173

[B4] Baldwin J. R. Reuben A. Newbury J. B. Danese A. (2019). Agreement between prospective and retrospective measures of childhood maltreatment: a systematic review and meta-analysis. JAMA Psychiatry. 10.1001/jamapsychiatry.2019.0097 [Epub ahead of print]. 30892562 PMC6551848

[B5] Bernstein D. P. Fink L. Handelsman L. Foote J. (1994). Childhood Trauma Questionnaire. A retrospective self-report: Manual. Harcourt Brace & Company.

[B6] Bernstein D. P. Stein J. A. Newcomb M. D. Walker E. Pogge D. Ahluvalia T.. (2003). Development and validation of a brief screening version of the Childhood Trauma Questionnaire. Child Abuse Neglect. 27, 169–190. 12615092 10.1016/s0145-2134(02)00541-0

[B7] Bersani F. S. Lindqvist D. Mellon S. H. Epel E. S. Yehuda R. Flory J.. (2016). Association of dimensional psychological health measures with telomere length in male war veterans. J. Affect. Disord. 190, 537–542. 10.1016/j.jad.2015.10.037 26571103

[B8] Blackburn E. H. (1991). Structure and function of telomeres. Nature 350, 569–573. 10.1038/350569a0 1708110

[B9] Blackburn E. H. (2000). Telomere states and cell fates. Nature 408, 53–56. 10.1038/35040500 11081503

[B10] Blackburn E. H. (2001). Switching and signaling at the telomere. Cell 106, 661–673. 10.1016/S0092-8674(01)00492-5 11572773

[B11] Blackburn E. H. (2005). Telomeres and telomerase: their mechanisms of action and the effects of altering their functions. FEBS Lett. 579, 859–862. 10.1016/j.febslet.2004.11.036 15680963

[B12] Blaze J. Asok A. Roth T. L. (2015). The long-term impact of adverse caregiving environments on epigenetic modifications and telomeres. Front. Behav. Neurosci. 9:79. 10.3389/fnbeh.2015.00079 25904853 PMC4389567

[B13] Blom E. H. Han L. Connolly C. Ho T. Lin J. LeWinn K. (2015). Peripheral telomere length and hippocampal volume in adolescents with major depressive disorder. Transl. Psychiatry 5:e676 10.1038/tp.2015.172 26556285 PMC5068765

[B14] Boks M. P. van Mierlo H. C. Rutten B. P. Radstake T. R. De Witte L. Geuze E.. (2015). Longitudinal changes of telomere length and epigenetic age related to traumatic stress and post-traumatic stress disorder. Psychoneuroendocrinology 51, 506–512. 10.1016/j.psyneuen.2014.07.011 25129579

[B15] Bremner J. D. Vermetten E. Mazure C. M. (2000). Development and preliminary psychometric properties of an instrument for the measurement of childhood trauma: the Early Trauma Inventory. Depress. Anxiety 12, 1–12. 10.1002/1520-6394(2000)12:1<1::AID-DA1>3.0.CO;2-W 10999240

[B16] Briere J. Kaltman S. Green B. L. (2008). Accumulated childhood trauma and symptom complexity. J. Trauma. Stress 21, 223–226. 10.1002/jts.20317 18404627

[B17] Brown D. W. Anda R. F. Tiemeier H. Felitti V. J. Edwards V. J. Croft J. B.. (2009). Adverse childhood experiences and the risk of premature mortality. Am. J. Prev. Med. 37, 389–396. 10.1016/j.amepre.2009.06.021 19840693

[B18] Cai N. Chang S. Li Y. Li Q. Hu J. Liang J.. (2015). Molecular signatures of major depression. Curr. Biol. 25, 1146–1156. 10.1016/j.cub.2015.03.008 25913401 PMC4425463

[B19] Cawthon R. M. Smith K. R. O'Brien E. Sivatchenko A. Kerber R. A. (2003). Association between telomere length in blood and mortality in people aged 60 years or older. Lancet 361, 393–395. 10.1016/S0140-6736(03)12384-7 12573379

[B20] Chen S. H. Epel E. S. Mellon S. H. Lin J. Reus V. I. Rosser R.. (2014). Adverse childhood experiences and leukocyte telomere maintenance in depressed and healthy adults. J. Affect. Disord. 169, 86–90. 10.1016/j.jad.2014.07.035 25173430 PMC4172492

[B21] Cloitre M. Garvert D. W. Brewin C. R. Bryant R. A. Maercker A. (2013). Evidence for proposed ICD-11 PTSD and complex PTSD: a latent profile analysis. Eur. J. Psychotraumatol. 4:20706. 10.3402/ejpt.v4i0.20706 23687563 PMC3656217

[B22] Cloitre M. Stolbach B. C. Herman J. L. van der Kolk B. Pynoos R. Wang J.. (2009). A developmental approach to complex PTSD: childhood and adult cumulative trauma as predictors of symptom complexity. J. Trauma. Stress 22, 399–408. 10.1002/jts.20444 19795402

[B23] Cook A. Spinazzola J. Ford J. Lanktree C. Blaustein M. Cloitre M. (2005). Complex trauma. Psychiatr. Ann. 35, 390–398. 10.3928/00485713-20050501-05

[B24] Dagan O. Asok A. Steele H. Steele M. Bernard K. (2017). Attachment security moderates the link between adverse childhood experiences and cellular aging. Dev. Psychopathol. 30, 1–13. 10.1017/S0954579417001705 29229013

[B25] D'Andrea W. Ford J. Stolbach B. Spinazzola J. van der Kolk B. A. (2012). Understanding interpersonal trauma in children: why we need a developmentally appropriate trauma diagnosis. Am. J. Orthopsychiatry 82, 187–200. 10.1111/j.1939-0025.2012.01154.x 22506521

[B26] Darrow S. M. Verhoeven J. E. Révész D. Lindqvist D. Penninx B. W. Delucchi K. L.. (2016). The association between psychiatric disorders and telomere length: a meta-analysis involving 14,827 persons. Psychosom. Med. 78:776. 10.1097/PSY.0000000000000356 27359174 PMC5003712

[B27] Drury S. S. Mabile E. Brett Z. H. Esteves K. Jones E. Shirtcliff E. A.. (2014). The association of telomere length with family violence and disruption. Pediatrics 134, e128–137. 10.1542/peds.2013-3415 24936002 PMC4067635

[B28] Dube S. R. Anda R. F. Felitti V. J. Chapman D. P. Williamson D. F. Giles W. H. (2001). Childhood abuse, household dysfunction, and the risk of attempted suicide throughout the life span: findings from the Adverse Childhood Experiences study. JAMA 286, 3089–3096. 10.1001/jama.286.24.3089 11754674

[B29] Dube S. R. Felitti V. J. Dong M. Giles W. H. Anda R. F. (2003). The impact of adverse childhood experiences on health problems: evidence from four birth cohorts dating back to 1900. Prev. Med. 37, 268–277. 10.1016/S0091-7435(03)00123-3 12914833

[B30] Epel E. S. Blackburn E. H. Lin J. Dhabhar F. S. Adler N. E. Morrow J. D.. (2004). Accelerated telomere shortening in response to life stress. Proc. Natl. Acad. Sci. USA. 101, 17312–17315. 10.1073/pnas.0407162101 15574496 PMC534658

[B31] Epel E. S. Crosswell A. D. Mayer S. E. Prather A. A. Slavich G. M. Puterman E.. (2018). More than a feeling: a unified view of stress measurement for population science. Front. Neuroendocrinol. 49, 146–169. 10.1016/j.yfrne.2018.03.001 29551356 PMC6345505

[B32] Epel E. S. Lithgow G. J. (2014). Stress biology and aging mechanisms: toward understanding the deep connection between adaptation to stress and longevity. J. Gerontol. Series A Biol. Sci. Med. Sci. 69, S10–S16. 10.1093/gerona/glu055 24833580 PMC4022128

[B33] Epel E. S. Prather A. A. (2018). Stress, telomeres, and psychopathology: toward a deeper understanding of a triad of early aging. Annu. Rev. Clin. Psychol. 14, 371–397. 10.1146/annurev-clinpsy-032816-045054 29494257 PMC7039047

[B34] Felitti V. J. Anda R. F. Nordenberg D. Williamson D. F. Spitz A. M. Edwards V.. (1998). Relationship of childhood abuse and household dysfunction to many of the leading causes of death in adults. The adverse childhood experiences (ACE). study. Am. J. Prev. Med. 14, 245–258. 10.1016/S0749-3797(98)00017-8 9635069

[B35] Foote J. Lovejoy M. (1995). Initial reliability and validity of the childhood trauma interview: a new multidimensional measure of childhood interpersonal trauma. Am. J. Psychiatry 152, 1329–1335. 10.1176/ajp.152.9.1329 7653689

[B36] Glass D. Parts L. Knowles D. Aviv A. Spector T. D. (2010). No correlation between childhood maltreatment and telomere length. Biol. Psychiatry 68, e21–e22. 10.1016/j.biopsych.2010.02.026 20678755 PMC2930212

[B37] Green J. G. McLaughlin K. A. Berglund P. A. Gruber M. J. Sampson N. A. Zaslavsky A. M.. (2010). Childhood adversities and adult psychiatric disorders in the national comorbidity survey replication I: associations with first onset of DSM-IV disorders. Arch. Gen. Psychiatry 67, 113–123. 10.1001/archgenpsychiatry.2009.186 20124111 PMC2822662

[B38] Greeson J. K. Briggs E. C. Kisiel C. L. Layne C. M. Ake G. S. 3rd Ko S. J.. (2011). Complex trauma and mental health in children and adolescents placed in foster care: findings from the national child traumatic stress network. Child Welfare 90:91. 22533044

[B39] Guarneri-White M. E. Arana A. A. Boyd E. Q. Jensen-Campbell L. A. (2018). It's more than skin-deep: the relationship between social victimization and telomere length in adolescence. Aggress. Behav. 44, 337–347. 10.1002/ab.21755 29484667

[B40] Hanssen L. M. Schutte N. S. Malouff J. M. Epel E. S. (2017). The relationship between childhood psychosocial stressor level and telomere length: a meta-analysis. Health Psychol. Res. 5:6378. 10.4081/hpr.2017.6378 28603779 PMC5452631

[B41] Hardt J. Rutter M. (2004). Validity of adult retrospective reports of adverse childhood experiences: review of the evidence. J. Child Psychol. Psychiatry 45, 260–273. 10.1111/j.1469-7610.2004.00218.x 14982240

[B42] Heim C. Binder E. B. (2012). Current research trends in early life stress and depression: review of human studies on sensitive periods, gene-environment interactions, and epigenetics. Exp. Neurol. 233, 102–111. 10.1016/j.expneurol.2011.10.032 22101006

[B43] Heim C. Shugart M. Craighead W. E. Nemeroff C. B. (2010). Neurobiological and psychiatric consequences of child abuse and neglect. Dev. Psychobiol. 52, 671–690. 10.1002/dev.20494 20882586

[B44] Honig L. S. Schupf N. Lee J. H. Tang M. X. Mayeux R. (2006). Shorter telomeres are associated with mortality in those with APOE ϵ4 and dementia. Ann. Neurol. 60, 181–187. 10.1002/ana.20894 16807921

[B45] Jodczyk S. Fergusson D. M. Horwood L. J. Pearson J. F. Kennedy M. A. (2014). No association between mean telomere length and life stress observed in a 30 year birth cohort. PLoS ONE 9:e97102. 10.1371/journal.pone.0097102 24816913 PMC4016252

[B46] Kananen L. Surakka I. Pirkola S. Suvisaari J. Lonnqvist J. Peltonen L.. (2010). Childhood adversities are associated with shorter telomere length at adult age both in individuals with an anxiety disorder and controls. PLoS ONE 5:e10826. 10.1371/journal.pone.0010826 20520834 PMC2876034

[B47] Kelly-Irving M. Mabile L. Grosclaude P. Lang T. Delpierre C. (2013). The embodiment of adverse childhood experiences and cancer development: potential biological mechanisms and pathways across the life course. Int. J. Public Health 58, 3–11. 10.1007/s00038-012-0370-0 22588310

[B48] Kessler R. C. McLaughlin K. A. Green J. G. Gruber M. J. Sampson N. A. Zaslavsky A. M.. (2010). Childhood adversities and adult psychopathology in the WHO World Mental Health Surveys. Br. J. Psychiatry 197, 378–385. 10.1192/bjp.bp.110.080499 21037215 PMC2966503

[B49] Kiecolt-Glaser J. K. Gouin J. P. Weng N. P. Malarkey W. B. Beversdorf D. Q. Glaser R. (2011). Childhood adversity heightens the impact of later-life caregiving stress on telomere length and inflammation. Psychosom. Med. 73, 16–22. 10.1097/PSY.0b013e31820573b6 21148804 PMC3051180

[B50] Kuffer A. L. O'Donovan A. Burri A. Maercker A. (2016). Posttraumatic stress disorder, adverse childhood events, and buccal cell telomere length in elderly swiss former indentured child laborers. Front. Psychiatry 7:147. 10.3389/fpsyt.2016.00147 27630582 PMC5005955

[B51] Lai T.-P. Wright W. E. Shay J. W. (2018). Comparison of telomere length measurement methods. Philos. Trans. R. Soc. B Biol. Sci. 373:20160451. 10.1098/rstb.2016.0451 29335378 PMC5784071

[B52] Levandowski M. L. Tractenberg S. G. de Azeredo L. A. De Nardi T. Rovaris D. L. Bau C. H.. (2016). Crack cocaine addiction, early life stress and accelerated cellular aging among women. Prog. Neuropsychopharmacol. Biol. Psychiatry 71, 83–89. 10.1016/j.pnpbp.2016.06.009 27346744

[B53] Li X. Wang J. Zhou J. Huang P. Li J. (2017a). The association between post-traumatic stress disorder and shorter telomere length: a systematic review and meta-analysis. J. Affect. Disord. 218, 322–326. 10.1016/j.jad.2017.03.048 28486180

[B54] Li Z. He Y. Wang D. Tang J. Chen X. (2017b). Association between childhood trauma and accelerated telomere erosion in adulthood: a meta-analytic study. J. Psychiatr. Res. 93(Suppl. C), 64–71. 10.1016/j.jpsychires.2017.06.002 28601667

[B55] Lindqvist D. Epel E. S. Mellon S. H. Penninx B. W. Revesz D. Verhoeven J. E.. (2015). Psychiatric disorders and leukocyte telomere length: Underlying mechanisms linking mental illness with cellular aging. Neurosci. Biobehav. Rev. 55, 333–364. 10.1016/j.neubiorev.2015.05.007 25999120 PMC4501875

[B56] Liu J. J. Wei Y. B. Forsell Y. Lavebratt C. (2017). Stress, depressive status and telomere length: does social interaction and coping strategy play a mediating role? J. Affect. Disord. 222, 138–145. 10.1016/j.jad.2017.07.007 28704801

[B57] Malan-Müller S. Hemmings S. M. J. Spies G. Kidd M. Fennema-Notestine C. Seedat S. (2013). Shorter telomere length-A potential susceptibility factor for HIV-associated neurocognitive impairments in South African woman. PLoS ONE 8:e58351 10.1371/journal.pone.0058351 23472184 PMC3589394

[B58] Mason S. M. Prescott J. Tworoger S. S. De Vivo I. Rich-Edwards J. W. (2015). Childhood physical and sexual abuse history and leukocyte telomere length among women in middle adulthood. PLoS ONE 10:e0124493. 10.1371/journal.pone.0124493 26053088 PMC4459951

[B59] Mather K. A. Jorm A. F. Parslow R. A. Christensen H. (2011). Is telomere length a biomarker of aging? A review. J. Gerontol. Series A Biol. Sci. Med. Sci. 66, 202–213. 10.1093/gerona/glq180 21030466

[B60] Mathur M. B. Epel E. Kind S. Desai M. Parks C. G. Sandler D. P.. (2016). Perceived stress and telomere length: a systematic review, meta-analysis, and methodologic considerations for advancing the field. Brain Behav. Immun. 54, 158–169. 10.1016/j.bbi.2016.02.002 26853993 PMC5590630

[B61] McFarland M. J. Taylor J. Hill T. D. Friedman K. L. (2017). Stressful life events in early life and leukocyte telomere length in adulthood. Adv. Life Course Res. 35, 37–45. 10.1016/j.alcr.2017.12.002

[B62] Mitchell A. M. Kowalsky J. M. Epel E. S. Lin J. Christian L. M. (2018). Childhood adversity, social support, and telomere length among perinatal women. Psychoneuroendocrinology 87, 43–52. 10.1016/j.psyneuen.2017.10.003 29035711 PMC5705286

[B63] Mitchell C. Hobcraft J. McLanahan S. S. Siegel S. R. Berg A. Brooks-Gunn J.. (2014). Social disadvantage, genetic sensitivity, and children's telomere length. Proc. Natl. Acad. Sci. USA. 111, 5944–5949. 10.1073/pnas.1404293111 24711381 PMC4000782

[B64] Moffitt T. E. the Klaus-Grawe Think Tank. (2013). Childhood exposure to violence and lifelong health: clinical intervention science and stress-biology research join forces. Dev. Psychopathol. 25(4 Pt 2), 1619–1634. 10.1017/S0954579413000801 24342859 PMC3869039

[B65] Montpetit A. J. Alhareeri A. A. Montpetit M. Starkweather A. R. Elmore L. W. Filler K.. (2014). Telomere length: a review of methods for measurement. Nurs. Res. 63, 289–299. 10.1097/NNR.0000000000000037 24977726 PMC4292845

[B66] O'Donovan A. Epel E. Lin J. Wolkowitz O. Cohen B. Maguen S.. (2011). Childhood trauma associated with short leukocyte telomere length in posttraumatic stress disorder. Biol. Psychiatry 70, 465–471. 10.1016/j.biopsych.2011.01.035 21489410 PMC3152637

[B67] Oliveira B. S. Zunzunegui M. V. Quinlan J. Batistuzzo de Medeiros S. R. Thomasini R. L. Guerra R. O. (2017). Lifecourse adversity and telomere length in older women from Northeast Brazil. Rejuvenation Res. 21, 294–303. 10.1089/rej.2017.1937 28482745

[B68] Oliveira B. S. Zunzunegui M. V. Quinlan J. Fahmi H. Tu M. T. Guerra R. O. (2016). Systematic review of the association between chronic social stress and telomere length: a life course perspective. Ageing Res. Rev. 26, 37–52. 10.1016/j.arr.2015.12.006 26732034

[B69] Osler M. Bendix L. Rask L. Rod N. H. (2016). Stressful life events and leucocyte telomere length: do lifestyle factors, somatic and mental health, or low grade inflammation mediate this relationship? results from a cohort of Danish men born in 1953. Brain Behav. Immun. 58, 248–253. 10.1016/j.bbi.2016.07.154 27470228

[B70] Price L. H. Kao H. T. Burgers D. E. Carpenter L. L. Tyrka A. R. (2013). Telomeres and early-life stress: an overview. Biol. Psychiatry 73, 15–23. 10.1016/j.biopsych.2012.06.025 22831981 PMC3495091

[B71] Puterman E. Gemmill A. Karasek D. Weir D. Adler N. E. Prather A. A.. (2016). Lifespan adversity and later adulthood telomere length in the nationally representative US Health and Retirement study. Proc. Natl. Acad. Sci. USA. 113, E6335–E6342. 10.1073/pnas.1525602113 27698131 PMC5081642

[B72] Revesz D. Milaneschi Y. Terpstra E. M. Penninx B. W. (2016). Baseline biopsychosocial determinants of telomere length and 6-year attrition rate. Psychoneuroendocrinology 67, 153–162. 10.1016/j.psyneuen.2016.02.007 26897704

[B73] Rich-Edwards J. W. Mason S. Rexrode K. Spiegelman D. Hibert E. Kawachi I.. (2012). Physical and sexual abuse in childhood as predictors of early onset cardiovascular events in women. Circulation 126, 920–927. 10.1161/CIRCULATIONAHA.111.076877 22787111 PMC3649533

[B74] Ridout K. K. Levandowski M. Ridout S. J. Gantz L. Goonan K. Palermo D.. (2017). Early life adversity and telomere length: a meta-analysis. Mol. Psychiatry 23, 858–871. 10.1038/mp.2017.26 28322278 PMC5608639

[B75] Ridout K. K. Ridout S. J. Price L. H. Sen S. Tyrka A. R. (2016). Depression and telomere length: a meta-analysis. J. Affect. Disord. 191, 237–247. 10.1016/j.jad.2015.11.052 26688493 PMC4760624

[B76] Ridout S. J. Ridout K. K. Kao H.-T. Carpenter L. L. Philip N. S. Tyrka A. R. (2015). Telomeres, early-life stress and mental illness, in Clinical Challenges in the Biopsychosocial Interface, eds Balon R. Wise T. N. (Basel: Karger Publishers), 92–108. 10.1159/000369088 PMC4476498 25832516

[B77] Riley G. Perrin M. Vaez-Azizi L. M. Ruby E. Goetz R. R. Dracxler R.. (2018). Telomere length and early trauma in schizophrenia. Schizophr Res. 199, 426–430. 10.1016/j.schres.2018.02.059 29618413 PMC8787687

[B78] Robles T. F. Carroll J. E. Bai S. Reynolds B. M. Esquivel S. Repetti R. L. (2016). Emotions and family interactions in childhood: Associations with leukocyte telomere length emotions, family interactions, and telomere length. Psychoneuroendocrinology 63, 343–350. 10.1016/j.psyneuen.2015.10.018 26551267 PMC5370166

[B79] Rosenthal R. (1995). Writing meta-analytic reviews. Psychol. Bull. 118:183 10.1037/0033-2909.118.2.183

[B80] Savolainen K. Eriksson J. G. Kananen L. Kajantie E. Pesonen A. K. Heinonen K.. (2014). Associations between early life stress, self-reported traumatic experiences across the lifespan and leukocyte telomere length in elderly adults. Biol. Psychol. 97, 35–42. 10.1016/j.biopsycho.2014.02.002 24530884

[B81] Schaakxs R. Verhoeven J. E. Oude Voshaar R. C. Comijs H. C. Penninx B. (2015). Leukocyte telomere length and late-life depression. Am. J. Geriatr. Psychiatry 23, 423–432. 10.1016/j.jagp.2014.06.003 25028345

[B82] Schaakxs R. Wielaard I. Verhoeven J. E. Beekman A. T. Penninx B. W. Comijs H. C. (2016). Early and recent psychosocial stress and telomere length in older adults. Int. Psychogeriatr. 28, 405–413. 10.1017/S1041610215001155 26265356

[B83] Schmid M. Petermann F. Fegert J. M. (2013). Developmental trauma disorder: pros and cons of including formal criteria in the psychiatric diagnostic systems. BMC Psychiatry 13:1. 10.1186/1471-244X-13-3 23286319 PMC3541245

[B84] Schutte N. S. Malouff J. M. (2014). The relationship between perceived stress and telomere length: a meta-analysis. Stress and Health. 32, 313–319. 10.1002/smi.2607 25393133

[B85] Schutte N. S. Malouff J. M. (2015). The association between depression AND leukocyte telomere length: a meta-analysis. Depress. Anxiety 32, 229–238. 10.1002/da.22351 25709105

[B86] Shalev I. (2012). Early life stress and telomere length: investigating the connection and possible mechanisms: a critical survey of the evidence base, research methodology and basic biology. Bioessays 34, 943–952. 10.1002/bies.201200084 22991129 PMC3557830

[B87] Shalev I. Moffitt T. E. Braithwaite A. W. Danese A. Fleming N. I. Goldman-Mellor S.. (2014). Internalizing disorders and leukocyte telomere erosion: a prospective study of depression, generalized anxiety disorder and post-traumatic stress disorder. Mol. Psychiatry 19, 1163–1170. 10.1038/mp.2013.183 24419039 PMC4098012

[B88] Shalev I. Moffitt T. E. Sugden K. Williams B. Houts R. M. Danese A.. (2013). Exposure to violence during childhood is associated with telomere erosion from 5 to 10 years of age: a longitudinal study. Mol. Psychiatry 18, 576–581. 10.1038/mp.2012.32 22525489 PMC3616159

[B89] Starkweather A. R. Alhaeeri A. A. Montpetit A. Brumelle J. Filler K. Montpetit M.. (2014). An integrative review of factors associated with telomere length and implications for biobehavioral research. Nurs. Res. 63, 36–50. 10.1097/NNR.0000000000000009 24335912 PMC4112289

[B90] Surtees P. G. Wainwright N. W. Pooley K. A. Luben R. N. Khaw K. T. Easton D. F.. (2011). Life stress, emotional health, and mean telomere length in the European Prospective Investigation into Cancer (EPIC)-Norfolk population study. J. Gerontol. A Biol. Sci. Med. Sci. 66, 1152–1162. 10.1093/gerona/glr112 21788649

[B91] Takubo K. Aida J. Izumiyama-Shimomura N. Ishikawa N. Sawabe M. Kurabayashi R. (2010). Changes of telomere length with aging. Geriatr. Gerontol. Int. (10 Suppl. 1), S197–206. 10.1111/j.1447-0594.2010.00605.x 20590834

[B92] Teicher M. H. Samson J. A. (2013). Childhood maltreatment and psychopathology: a case for ecophenotypic variants as clinically and neurobiologically distinct subtypes. Am. J. Psychiatry 170, 1114–1133. 10.1176/appi.ajp.2013.12070957 23982148 PMC3928064

[B93] Teicher M. H. Samson J. A. Anderson C. M. Ohashi K. (2016). The effects of childhood maltreatment on brain structure, function and connectivity. Nat. Rev. Neurosci. 17, 652–666. 10.1038/nrn.2016.111 27640984

[B94] Tomasdottir M. O. Sigurdsson J. A. Petursson H. Kirkengen A. L. Krokstad S. McEwen B.. (2015). Self reported childhood difficulties, adult multimorbidity and allostatic load. A cross-sectional analysis of the Norwegian HUNT study. PLoS ONE 10:e0130591. 10.1371/journal.pone.0130591 26086816 PMC4472345

[B95] Tyrka A. R. Parade S. H. Price L. H. Kao H. T. Porton B. Philip N. S.. (2016). Alterations of mitochondrial DNA copy number and telomere length with early adversity and psychopathology. Biol. Psychiatry 79, 78–86. 10.1016/j.biopsych.2014.12.025 25749099 PMC4503518

[B96] Tyrka A. R. Price L. H. Kao H. T. Porton B. Marsella S. A. Carpenter L. L. (2010). Childhood maltreatment and telomere shortening: preliminary support for an effect of early stress on cellular aging. Biol. Psychiatry 67, 531–534. 10.1016/j.biopsych.2009.08.014 19828140 PMC2853238

[B97] van der Kolk B. A. Pynoos R. S. Cicchetti D. Cloitre M. D'Andrea W. Ford J. D. (2009). Proposal to Include a Developmental Trauma Disorder Diagnosis for Children and Adolescents in DSM-V. Unpublished manuscript.

[B98] van Ockenburg S. L. Bos E. H. de Jonge P. van der Harst P. Gans R. O. Rosmalen J. G. (2015). Stressful life events and leukocyte telomere attrition in adulthood: a prospective population-based cohort study. Psychol. Med. 45, 2975–2984. 10.1017/S0033291715000914 26219269

[B99] Verhoeven J. E. van Oppen P. Puterman E. Elzinga B. Penninx B. W. (2015). The association of early and recent psychosocial life stress with leukocyte telomere length. Psychosom. Med. 77, 882–891. 10.1097/PSY.0000000000000226 26374947

[B100] Vincent J. Hovatta I. Frissa S. Goodwin L. Hotopf M. Hatch S. L.. (2017). Assessing the contributions of childhood maltreatment subtypes and depression case-control status on telomere length reveals a specific role of physical neglect. J. Affect. Disord. 213, 16–22. 10.1016/j.jad.2017.01.031 28187293 PMC6191534

[B101] Widom C. S. (1999). Posttraumatic stress disorder in abused and neglected children grown up. Am. J. Psychiatry 156, 1223–1229. 10450264 10.1176/ajp.156.8.1223

[B102] Widom C. S. Czaja S. J. Dutton M. A. (2008). Childhood victimization and lifetime revictimization. Child Abuse Negl. 32, 785–796. 10.1016/j.chiabu.2007.12.006 18760474 PMC2572709

[B103] Widom C. S. DuMont K. Czaja S. J. (2007). A prospective investigation of major depressive disorder and comorbidity in abused and neglected children grown up. Arch. Gen. Psychiatry 64, 49–56. 10.1001/archpsyc.64.1.49 17199054

[B104] Willeit P. Willeit J. Mayr A. Weger S. Oberhollenzer F. Brandstatter A.. (2010). Telomere length and risk of incident cancer and cancer mortality. JAMA 304, 69–75. 10.1001/jama.2010.897 20606151

[B105] Zalli A. Carvalho L. A. Lin J. Hamer M. Erusalimsky J. D. Blackburn E. H.. (2014). Shorter telomeres with high telomerase activity are associated with raised allostatic load and impoverished psychosocial resources. Proc. Natl. Acad. Sci. USA. 111, 4519–4524. 10.1073/pnas.1322145111 24616496 PMC3970484

