# Supplementary material for: Adverse Childhood Experiences and Telomere Length a Look Into the Heterogeneity of Findings—A Narrative Review
Source: Front Neurosci. 2019 May 22;13:490. doi: 10.3389/fnins.2019.00490 (PMC6541108; doi:10.3389/fnins.2019.00490)
Supplement: Supplementary file 1 [file Data_Sheet_1.PDF]

**Supplementary Material**

Table 1. Supplementary Characteristics

| Author (Year) | Type of Adversities | Conclusions |
|---|---|---|
| (Bersani et al., 2016) | "Exposure to early life trauma was evaluated using the Early Trauma Inventory (ETI)–Self Report Short Form "(p.538) | "Across subjects, TL was negatively correlated with early trauma." (p.537) "These preliminary findings suggest that early trauma, severity of perceived stress and general psychopathological symptoms are more closely associated with shorter TL than is the severity of core diagnostic symptoms of PTSD or MDD, whereas positive affect is associated with longer TL." (p.537) |
| (Blom et al., 2015) | CM; CTQ Subscales: emotional/physical/sexual abuse, emotional/physical neglect; The CTQ was collected in a subsample, to test for an association between childhood trauma and TL shortening in adolescent depression. | TL was not associated with CTQ total score in either group. "Although significantly higher CTQ scores were found in the MDD sample compared with the HC, no associations were found between CTQ-score and TL […] within the MDD sample. " (p.5) |
| (Boks et al., 2015) | "Traumatic experiences during childhood were assessed using the 27-item Dutch version of the self-report version of the Early Trauma Inventory (ETI). This questionnaire assesses exposure to potentially traumatic experiences before the age of 18 years (general trauma, physical abuse, emotional abuse and sexual abuse)." (p.507) | "No association between childhood trauma measure and TL, nor trauma and TL. The Addition of childhood trauma measure to the analysis of TL and DNAm age showed no association of pre-deployment trauma with change in TL or DNAm age, nor was it associated with these outcome measures at baseline." (p.510) |
| (Cai et al., 2015) | "Stressful life events and childhood sexual abuse were assessed retrospectively. The stressful life events section of the CONVERGE interview was developed for the Virginia Adult Twin Study of Psychiatric and Use Disorders (VATSPUD). It assesses 16 traumatic life-time events and the age at their occurrence." (p.1152) | "TL was significantly shorter in those who had experienced more stressful life events […] and in those reporting childhood sexual abuse […]." (p.1148) |
| (Chen et al., 2014) | "Subjects reported on Adverse Childhood Experiences (ACE) using the self- administered 8-item Adverse Childhood Experiences scale. This scale assesses history of personal abuse, neglect, and household dysfunction. Scores range from zero (no history of ACEs) to eight (all eight types of ACEs)." (p.87) | "In healthy controls, greater ACE exposure was associated with shorter LTL but was unassociated with telomerase activity. In MDD, however, the opposite pattern was seen: greater ACE exposure was unrelated to LTL but was associated with increased telomerase activity and with a higher telomerase: LTL ratio." (p.86) |
| (Dagan et al., 2017) | "The ACE instrument assesses for 10 ACE, including 5 that reflect experiences of maltreatment (i.e., physical abuse, physical neglect, emotional abuse, emotional neglect, and sexual abuse) and 5 that reflect experiences of household dysfunction (i.e., parent with psychopathology, incarcerated parent, parent with substance abuse problems, parental divorce/ separation, and domestic violence)."(p.4) | "Significant interaction between attachment state of mind and ACE in predicting telomere length. Whereas the association between number of ACE and telomere length was nonsignificant for secure–autonomous, […], and insecure–preoccupied young adults, […], there was a strong negative association between number of ACE and telomere length for insecure–dismissing young adults […]." (p.1) |
| (Drury et al., 2014) | Cumulative family instability (witnessing family violence, family suicide, and incarceration); life events were categorized as present or absent; cumulative family in- stability was classified as both a continuous variable (0, 1, or 2+ events) and as high (>1 event) compared with low (no events); parents were interviewed | Cumulative exposure to interpersonal violence and family disruption correlated with TL. Gender moderated these associations (only in girls). |
| (Glass et al., 2010) | Childhood maltreatment (CM); Physical/sexual abuse; One questionnaire asked specifically about maltreatment in childhood and one referred to maltreatment at any time. | TL did not differ between subjects with and without experiences of abuse. CM was not linked to TL. |
| (Guarneri-White et al., 2018) | "Two common measures of victimization were used for this study. The Direct and Indirect Aggression Scale—Victim Version (DIAS-VS) […] examines the frequency of three types of peer victimization: indirect, physical, and verbal. The Children's Social Experiences Questionnaire—Self-Report […] assesses peer victimization through questions about relational and overt aggression. Both the DIAS-VS and the CSEQ-SR have evidenced high reliability[…]."(p.3) | "Greater instances of being socially, but not physically, victimized were associated with shorter telomeres, as well as more frequent and severe health complaints."(p.1) |

| | | |
|---|---|---|
| (Jodczyk et al., 2014) | Exposure to abuse (sexual, physical), violence and neglect during childhood before the age of 16. Sexual abuse was assessed using retrospective reports obtained at ages 18 and 21 years; Child physical abuse (CPA) was assessed on the basis of retrospective reports obtained at ages 18 and 21 of the extent to which the individual's parents used phys(McFarland et al., 2017)(McFarland et al., 2017)(McFarland et al., 2017)McFarland (2017)ical punishment during their childhood; At age 16 participants completed the parental care scale of the Parental Bonding Instrument (PBI); Selected series of items from the Conflict Tactics Scale (CTS) to assess the extent to which they reported witnessing incidents of inter-parental violence; overall measure of exposure. | "No associations were found between telomere length measured at age 28–30 years and life course adversity or stress for specific measures and for the summary risk scores for each developmental domain […]. Our results in this well- studied birth cohort do not support prior reports of such associations, and underscore the need for more extensive replication of proposed links between stress and telomere biology […]." (p.1) |
| (Kananen et al., 2010) | Adverse social environment in childhood, including financial difficulties, parental unemployment, parental physical/mental illness, familial conflict, bullying, personal illness. | Shorter TL associated with a greater number of childhood adverse life event in both, subjects with and without anxiety disorder. |
| (Kiecolt-Glaser et al., 2011) | CM; CTQ subscales: Emotional/physical/sexual abuse, emotional/physical neglect;<br>Childhood adversity (parental death, parental marital conflict, familial mental illness, familial alcohol problems, lack of close relationship with adult) | Shorter telomeres associated with multiple childhood adversities (2 or more childhood adversities). Telomere difference equivalent to 7 to 15 years of shortened lifespan. Abuse without other adversities was not associated with TL. |
| (Kuffer et al., 2016) | "The Childhood Trauma Questionnaire (CTQ) was used to assess childhood trauma exposure. The Childhood Trauma Questionnaire – Short Form (CTQ-SF) is a 28-item self-report inventory that provides brief and reliable screening for histories of abuse and neglect; emotional, physical, and sexual abuse, and emotional and physical neglect." (p.3) | "Results revealed a trend toward longer BTL in participants with partial or full PTSD, and longer BTL was marginally associated with higher CTQ scores. Within-group analyses indicated no significant association between BTL and CTQ scores." (p.1) |
| (Levandowski et al., 2016) | CTQ Subscales: emotional/physical/sexual abuse, emotional/physical neglect | "The current study provides a new evidence of shorter TL in crack cocaine dependent-women in comparison with elderly women. Moreover, this study demonstrates that ELS has an effect on telomere." (p.87) |
| (Liu et al., 2017) | "Childhood adversity (CA) before the age of 18 years was assessed in wave 1 by three questions. "Did any of your parents die?" If the answer was no, then scored as 1; if yes, scored as 2. For the questions "Did your family have financial problems?" and "Did friction exist in your family?", a three-point Likert scale was used from 1 (no) to 3 (2 = yes, milder or shorter periods, 3 = yes, more difficult or longer periods). The childhood adversity variable used was the sum score of these three items, thus a sum score of 3 meant no childhood adversity." (p.139) | "The key variables were correlated in expected directions. In females, depressive status and age had direct negative effects on TL ($p < 0.05$) and both CA ($p = 0.025$) and NLE ($p < 0.003$) had indirect negative effects on TL. For males, the effects of stressors and depressive status on TL were mediated by social interaction ($p = 0.005$) and the coping strategy worry ($p = 0.005$). In females, no mediation effect of social interaction and coping strategy was detected." (p.138) |
| (Malan-Müller et al., 2013) | Childhood trauma included emotional, physical, and sexual abuse as well as emotional and physical neglect (28 items) | HIV-positive individuals had significantly shorter LTL compared to HIV-negative individuals. No significant interaction effect between HIV status and childhood trauma on LTL. No significant association between relative LTL and childhood trauma. |
| (Mason et al., 2015) | "Child/adolescent physical abuse was assessed using questions from the Revised Conflict Tactics Scale, which asked participants to report the frequency with which a parent, step-parent or adult guardian pushed, grabbed, or shoved; kicked, bit, or punched; hit with something that hurt; choked or burned; or physically attacked the participant when she was a child (age 0–10) or adolescent (age 11–17)." (p.3) "Sexual Experiences Survey (SES) was assessed; categories: none, sexual touching only, and forced sexual activity." (p.4) | "Reduction in TL associated with moderate physical abuse versus no physical abuse, but there was no evidence of a dose-response relationship for increased severity of physical abuse. No associations were noted for sexual abuse. No evidence of an association between severity of childhood physical or sexual abuse and LTL in the NHSII." (p.1) |

| | | |
|---|---|---|
| (McFarland et al., 2017) | "Most SLE indices include indicators for sexual abuse, physical abuse, emotional abuse, witnessing abuse, having a parent with a drinking or drug problem, or having parents that divorced." (p.39); (32 Items) | "The complete SLEs index was inversely associated with telomere length (b=−0.007; p=0.002) for those adults under the age of 45. Each additional SLE experienced before the age of 18 was associated with a reduction in TL equivalent to one additional year of age. The association between the complete SLEs index and TL for those under the age of 45 was statistically different from those aged 45 or older." (p.37) |
| (Mitchell et al., 2018) | Childhood trauma included emotional, physical, and sexual abuse as well as emotional and physical neglect; (28 items) | "No significant effects on telomere length were observed in relation to emotional abuse (p= 0.82), physical abuse (p= 0.49), sexual abuse (p= 0.34), emotional neglect (p= 0.22) and physical neglect (p = 0.92)." (p. 47) |
| (Mitchell et al., 2014) | High levels of poverty, low levels of maternal education, high levels of family instability, harsh parenting, and maternal depression. "To measure harsh parenting, a count of how often mothers engaged in harsh psychological behaviors (e.g., yelling, threatening) and harsh physical behaviors (hitting, slapping) was used. These items were taken from the Conflict Tactics Scale." (p.5947) | African American boys who grow up in highly disadvantaged environments have shorter telomeres (at age 9) than boys who grow up in highly advantaged environments. Significant associations between low income, low maternal education, unstable family structure, and harsh parenting and TL. |
| (O'Donovan et al., 2011) | Physical neglect, family violence, physical abuse, forced sexual touch, or forced sexual intercourse; 5 items; "Participants were asked if they had been exposed to any of the following experiences to the extent that they felt that they could die or be physically harmed, physical neglect, family violence, physical abuse, forced sexual touch, or forced sexual intercourse at or before age 14." (p.466) | "Exposure to childhood trauma was also associated with short LTL. In fact, childhood trauma appeared to account for the PTSD group difference in LTL; only participants with PTSD and exposure to multiple categories of childhood trauma had significantly shorter LTL than controls." (p.465) |
| (Oliveira et al., 2017) | "These questions included the following: having witnessed physical family violence, experienced physical abuse by a close relative, hunger, poor economic status, prolonged parental unemployment, parental death, divorce of parents, and parental abuse of alcohol. The response was dichotomous (yes/no) and one ''point'' given for each category of self-reported adverse experience." (p.2); (8 Items) | "Women with low education had been exposed to more ACEs, and among them those experiencing two or more ACEs had longer TL than women exposed to ≤1 ACEs (p = 0.03)." (p.1) |
| (Osler et al., 2016) | "Psychosocial stress in childhood included six types of events: Prolonged parental illness; Death of a parent; Being placed away from home; Longstanding family conflicts; Longstanding parental unemployment; and Long-term parental financial problems." (p.249)<br>Overall psychosocial stress was created using an overall index of psychosocial stress in childhood, in work life and in adulthood. | "Total number of stressful events experienced during the life course was not associated with TL. In terms of sensitive periods, we found that number of stressful events in childhood was associated with shorter TL. This relation was particularly strong for being placed away from home." (p.248) |
| (Puterman et al., 2016) | "Childhood adversity included whether before the age of 18, the respondent's (i) family received help from relatives because of financial difficulties, (ii) family ever had to relocate due to financial difficulties, (iii) father ever lost his job, and (iv) parents' substance or alcohol use caused problems in the home. Childhood adversity also included whether the respondent (v) had ever experienced physical abuse before age 18, (vi) had to repeat a year of school, and (vii) had gotten into trouble with police. The first three childhood adversity items are considered financial in nature, whereas the final four are social or traumatic." (p.E6337) | "Single adversities tended to have nonsignificant relations with TL. Lifetime cumulative adversity predicted 6% greater odds of shorter TL. This result was mainly due to childhood adversity. In adjusted models for cumulative childhood adversity, the occurrence of each additional childhood event predicted 11% increased odds of having short telomeres. This result appeared mainly because of social/traumatic exposures rather than financial exposures." (p. E6335) |
| (Revesz et al., 2016) | "Childhood trauma was assessed with the Childhood Trauma Interview (CTI), in which participants were asked whether they were emotionally neglected, psychologically abused, physically abused or sexually abused before the age of 16. The CTI reports the sum of the categories that were scored from 0 to 2 (0: never happened; 1: sometimes; 2: happened regularly), resulting in an index score between 0 and 8." (p.155) | "Childhood trauma was not associated with current TL. […] However, childhood trauma did predict telomere attrition, especially when multiple traumatic events are reported." (p.160) |

| | | |
|---|---|---|
| (Riley et al., 2018) | General traumatic events, as well as physical, sexual and emotional abuse. | "While the trauma severity was not significantly associated with the mean group-level LTL in cases or controls, there were significant sex differences regarding these associations in both groups. Only male schizophrenia cases demonstrated the hypothesized significant LTL shortening from early trauma. Conversely, only female controls showed this same effect." (p.2-3) |
| (Robles et al., 2016) | Parent-child interactions: Items were based on the Youth Everyday Social Interaction and Mood measure. Child-reported parent-child conflict and warmth. Marital interactions: Items were based on the Child Home Data Questionnaire. Daily mood: Child-reported negative (sad, mean, unhappy, tense, angry, worried) and positive mood (lively, happy, relaxed, full of energy, cheerful, calm, proud, loved). (see p.348) | "Among children aged 8-13 years, a stronger association between negative mood and marital conflict, suggesting greater negative mood reactivity to marital conflict, was related to shorter LTL." (p. 343) |
| (Savolainen et al., 2014) | Evacuation to temporary foster care unaccompanied by parents (Finnish National Archives' register). Trauma area questions from the Traumatic Experiences Checklist (TEC). Emotional traumas, three questions on emotional neglect and three questions on emotional abuse. Physical traumas, three questions on physical abuse and three questions on threat to life/pain/bizarre punishment. | "Results suggest that while ELS or self-reported traumatic experiences are not per se associated with LTL measured decades later, ELS may in combination with self-reported traumatic events be associated with accelerated biological aging." (p.35) |
| (Schaakxs et al., 2016) | "Using the Childhood Trauma Inventory participants were asked whether they experienced any of the following four types of childhood abuse before age 16. […]. The sum of the final frequency scores per type of abuse was used to create a childhood trauma index (range 0–8) to indicate the cumulative exposure to childhood abuse." (p.407)<br>"Childhood adverse events were measured by asking participants whether they were separated from their parents during childhood […], whether their parents were divorced, and whether one of their parents had died, all before the age of 16." (p.407) | "Childhood abuse, recent negative life events and loneliness were unrelated to TL. Only having experienced any childhood adverse event was weakly but significantly negatively associated with TL." (p.405) |
| (Shalev et al., 2013) | Exposure to domestic violence between mother and her partner (CTS), frequent bullying victimization (interview), physical maltreatment by an adult (standardized clinical interview protocol); Interviews with mother; at Children's age of 5, 7 and 10; over all assessments, index of cumulative violence exposure | "Children who experienced two or more kinds of violence exposure showed significantly more telomere erosion between age-5 baseline and age-10 follow-up measurements" (p.576).<br>Cumulative violence exposure is associated with accelerated TL erosion in children. |
| (Shalev et al., 2014) | "Measures of CM in the Dunedin study included: (1) maternal rejection assessed at age 3 years by observational ratings of mothers' interaction with the study children, (2) harsh discipline assessed at ages 7 and 9 years by parental report of disciplinary behaviors, (3) exposure to disruptive caregiver changes assessed through age 11 and defined by two or more changes of the child's primary caregiver, (4) exposure to physical abuse, and (5) exposure to sexual abuse assessed retrospectively at age 26 years. The number of indicators was summed to a scale of 0 to 2+. " (p.1165) | No association between CM and LTL at age 38 years.<br>"Analyses showed that the persistence of internalizing disorders across repeated assessments from ages 11 to 38 years predicted shorter LTL at age 38 years in a dose–response manner, specifically in men […]" (p.1163)<br>"Additional analyses using DNA from blood collected at two time points (ages 26 and 38 years) showed that LTL erosion was accelerated among men who were diagnosed with internalizing disorder in the interim […]." (p.1163) |
| (Surtees et al., 2011) | "Social adversity included 8 specific (potentially) traumatic circumstances experienced prior to age 17 years (separation from their mother for more than 1 year, hospital stay for 2 or more weeks, parental divorce, parental unemployment, an experience that was so frightening as to be thought about for years, being sent away from home because of doing something wrong, parental alcohol or drug use, and experience of physical abuse)." (p.1153) | Shorter telomeres associated with social adversities (greater number of adverse events). Each additional childhood difficulty reported was equivalent to an approximate 3-year increase in aging. |

| | | |
|---|---|---|
| (Tyrka et al., 2010) | CM; CTQ Subscales: Emotional/physical/sexual abuse, emotional/physical neglect | Shorter telomeres associated with CM, results suggested that neglect may have the most robust effect. |
| (Tyrka et al., 2016) | "Loss of a parent before age 18 included death and/or prolonged separation/desertion (i.e., parent deserted for at least 6 months with no attempts at contact or responses to child's attempts).<br>The Childhood Trauma Questionnaire; moderate-severe levels of one or more of the five types of maltreatment (physical, sexual, and emotional abuse and physical and emotional neglect) were assessed." (p.80) | "Childhood adversity and lifetime psychopathology were each associated with shorter telomeres and higher mtDNA copy numbers. Significantly higher mtDNA copy numbers and shorter telomeres were seen in individuals with major depression, depressive disorders, and anxiety disorders, as well as those with parental loss and CM." (p.78) |
| (van Ockenburg et al., 2015) | Life events were assessed with an adjusted version of the List of Threatening Events (LTE). Three exposure variables: 'childhood LTE score' (number of different adverse life events before age 12), 'recent LTE score' (number of different adverse life events in previous year), and 'lifetime LTE score' (number of different adverse life events in all completed age groups). | "Recent life events significantly predicted telomere attrition prospectively. No significant cross-sectional relationship between the lifetime LTE score and TL. Nor was found that exposure to adverse life events before the age of 12 is associated with TL in adulthood." (p.2975) |
| (Verhoeven et al., 2015) | "Childhood life events were assessed retrospectively and included three categories of life events: a) divorce of parents, b) parental loss, and c) separation from home (placed in a juvenile prison, raised in a foster family, and placed in a child home) before age 16 years." (p.884)<br>"Childhood trauma was assessed with the Childhood Trauma Interview (CTI). Participants were asked whether they were emotionally neglected, psychologically abused, physically abused, or sexually abused before the age of 16 years." (p.884) | "Childhood life events and childhood trauma were not related to shorter TL. However, we found negative associations between recent stressful life events and TL. Persons had shorter telomeres if they reported more stressful life events in the past year and 1 to 5 years ago." (p.882)<br>"Adversities that happened earlier in life, specifically childhood life events or trauma, were unrelated to TL in adulthood in the current study." (p.887) |
| (Vincent et al., 2017) | Childhood trauma included emotional, physical, and sexual abuse as well as emotional and physical neglect; (28 items) | "Four subtypes of childhood maltreatment also demonstrated no significant main effect on RTL, however a history of physical neglect did significantly predict shorter RTL in adulthood (F (1,174) =7.559, p=0.007, q=0.042, Variance Explained=4.2%), which was independent of case/control status. RTL was further predicted by severity of physical neglect, with the greatest differences observed in older maltreated individuals ( > 50 years old)." (p.16) |
| (Zalli et al., 2014) | Early Life Adversity: experienced the death of a parent or sibling before age 16; had been separated from their parents for at least 12 months in childhood; and lived in a family in which one member had severe mental illness (including depression), drug problems, or chronic illness. | "The Proportion reporting that their mothers had died before they were 16 y of age was greater in the short TL/high TA group (13.2%) than in the long TL (6.0%) or short TL/low TA (2.4%) group. Compared with respondents in the long TL group, the odds of having experienced such maternal loss were significantly greater in the short TL/high TA group." (p.4521) |

**Abbreviations:** ACE: Adverse Childhood Experiences; BMI: body mass index; BP: blood pressure; BTL: buccal cell telomere length; CA: childhood Adversity; CM: childhood maltreatment; CPA: child physical abuse; CSEQ: children's social experiences questionnaire; CTI: childhood trauma interview; CTQ: childhood trauma questionnaire; CTS: conflict tactics scale; DIAS-VS: direct and indirect aggression scale-victim version; DNA: deoxyribonucleic acid; DNAm age: DNA methylation age; ELD: elderly people; ELS: early life stress; ETI: early trauma inventory; Exp.: exposed; f: female; HIV: human immunodeficiency virus; HC: healthy controls; HLEQ: health and life experiences questionnaire; int. dis.: internalizing disorders; LHC: life history calendar; LSC: life stressor checklist; LTE: list of threatening events; LTL: leukocyte telomere length; m: male; MDD: major depressive disorder; mod.: modified; N: number of participants; NA: not available; NLE: adulthood negative life events; PBI: parental bonding instrument; PAPA: preschool age psychiatric assessment; PBMC: peripheral blood mononuclear cells; PTSD: posttraumatic stress disorder; qPCR: quantitative polymerase chain reaction; SES: socioeconomic status; SF: short form; SR: self-report; SLE: stressful life events in early life; TA: Telomerase Activity; TEC: traumatic experiences checklist; TL: Telomere length; UK: United Kingdom; USA: United States of America

Bersani, F.S., Lindqvist, D., Mellon, S.H., Epel, E.S., Yehuda, R., Flory, J., et al. (2016). Association of dimensional psychological health measures with telomere length in male war veterans. *J Affect Disord* 190**,** 537-542. doi: 10.1016/j.jad.2015.10.037.
Blom, E.H., Han, L., Connolly, C., Ho, T., Lin, J., LeWinn, K., et al. (2015). Peripheral telomere length and hippocampal volume in adolescents with major depressive disorder. *Translational psychiatry* 5(11)**,** e676.
Boks, M.P., van Mierlo, H.C., Rutten, B.P., Radstake, T.R., De Witte, L., Geuze, E., et al. (2015). Longitudinal changes of telomere length and epigenetic age related to traumatic stress and post-traumatic stress disorder. *Psychoneuroendocrinology* 51**,** 506-512. doi: 10.1016/j.psyneuen.2014.07.011.
Cai, N., Chang, S., Li, Y., Li, Q., Hu, J., Liang, J., et al. (2015). Molecular signatures of major depression. *Curr Biol* 25(9)**,** 1146-1156. doi: 10.1016/j.cub.2015.03.008.
Chen, S.H., Epel, E.S., Mellon, S.H., Lin, J., Reus, V.I., Rosser, R., et al. (2014). Adverse childhood experiences and leukocyte telomere maintenance in depressed and healthy adults. *J Affect Disord* 169**,** 86-90. doi: 10.1016/j.jad.2014.07.035.
Dagan, O., Asok, A., Steele, H., Steele, M., and Bernard, K. (2017). Attachment security moderates the link between adverse childhood experiences and cellular aging. *Development and psychopathology***,** 1-13.
Drury, S.S., Mabile, E., Brett, Z.H., Esteves, K., Jones, E., Shirtcliff, E.A., et al. (2014). The association of telomere length with family violence and disruption. *Pediatrics* 134(1)**,** e128-137. doi: 10.1542/peds.2013-3415.
Glass, D., Parts, L., Knowles, D., Aviv, A., and Spector, T.D. (2010). No correlation between childhood maltreatment and telomere length. *Biological psychiatry* 68(6)**,** e21-e22.
Guarneri-White, M.E., Arana, A.A., Boyd, E.Q., and Jensen-Campbell, L.A. (2018). It's more than skin-deep: The relationship between social victimization and telomere length in adolescence. *Aggressive behavior*.
Jodczyk, S., Fergusson, D.M., Horwood, L.J., Pearson, J.F., and Kennedy, M.A. (2014). No association between mean telomere length and life stress observed in a 30 year birth cohort. *PLoS One* 9(5)**,** e97102. doi: 10.1371/journal.pone.0097102.
Kananen, L., Surakka, I., Pirkola, S., Suvisaari, J., Lonnqvist, J., Peltonen, L., et al. (2010). Childhood adversities are associated with shorter telomere length at adult age both in individuals with an anxiety disorder and controls. *PLoS One* 5(5)**,** e10826. doi: 10.1371/journal.pone.0010826.
Kiecolt-Glaser, J.K., Gouin, J.P., Weng, N.P., Malarkey, W.B., Beversdorf, D.Q., and Glaser, R. (2011). Childhood adversity heightens the impact of later-life caregiving stress on telomere length and inflammation. *Psychosom Med* 73(1)**,** 16-22. doi: 10.1097/PSY.0b013e31820573b6.
Kuffer, A.L., O'Donovan, A., Burri, A., and Maercker, A. (2016). Posttraumatic Stress Disorder, Adverse Childhood Events, and Buccal Cell Telomere Length in Elderly Swiss Former Indentured Child Laborers. *Front Psychiatry* 7**,** 147. doi: 10.3389/fpsyt.2016.00147.
Levandowski, M., Tractenberg, S., Rizzo, L., Maurya, P., Brietzke, E., De Nardi, T., et al. (2016). Telomere Length in Crack/Cocaine Use Disorder with Early Life Stress. *Neuropsychopharmacology* 40, S592-S593.
Liu, J.J., Wei, Y.B., Forsell, Y., and Lavebratt, C. (2017). Stress, depressive status and telomere length: Does social interaction and coping strategy play a mediating role? *J Affect Disord* 222**,** 138-145. doi: 10.1016/j.jad.2017.07.007.
Malan-Müller, S., Hemmings, S.M.J., Spies, G., Kidd, M., Fennema-Notestine, C., and Seedat, S. (2013). Shorter telomere length-A potential susceptibility factor for HIV-associated neurocognitive impairments in South African woman. *PLoS One* 8(3)**,** e58351.
Mason, S.M., Prescott, J., Tworoger, S.S., De Vivo, I., and Rich-Edwards, J.W. (2015). Childhood Physical and Sexual Abuse History and Leukocyte Telomere Length among Women in Middle Adulthood. *PLoS One* 10(6)**,** e0124493. doi: 10.1371/journal.pone.0124493.
McFarland, M.J., Taylor, J., Hill, T.D., and Friedman, K.L. (2017). Stressful Life Events in Early Life and Leukocyte Telomere Length in Adulthood. *Advances in Life Course Research*.
Mitchell, A.M., Kowalsky, J.M., Epel, E.S., Lin, J., and Christian, L.M. (2018). Childhood adversity, social support, and telomere length among perinatal women. *Psychoneuroendocrinology* 87**,** 43-52.
Mitchell, C., Hobcraft, J., McLanahan, S.S., Siegel, S.R., Berg, A., Brooks-Gunn, J., et al. (2014). Social disadvantage, genetic sensitivity, and children's telomere length. *Proc Natl Acad Sci U S A* 111(16)**,** 5944-5949. doi: 10.1073/pnas.1404293111.
O'Donovan, A., Epel, E., Lin, J., Wolkowitz, O., Cohen, B., Maguen, S., et al. (2011). Childhood trauma associated with short leukocyte telomere length in posttraumatic stress disorder. *Biol Psychiatry* 70(5)**,** 465-471. doi: 10.1016/j.biopsych.2011.01.035.
Oliveira, B.S., Zunzunegui, M.V., Quinlan, J., Batistuzzo de Medeiros, S.R., Thomasini, R.L., and Guerra, R.O. (2017). Lifecourse Adversity and Telomere Length in Older Women from Northeast Brazil. *Rejuvenation Res*. doi: 10.1089/rej.2017.1937.
Osler, M., Bendix, L., Rask, L., and Rod, N.H. (2016). Stressful life events and leucocyte telomere length: Do lifestyle factors, somatic and mental health, or low grade inflammation mediate this relationship? Results from a cohort of Danish men born in 1953. *Brain Behav Immun* 58**,** 248-253. doi: 10.1016/j.bbi.2016.07.154.
Puterman, E., Gemmill, A., Karasek, D., Weir, D., Adler, N.E., Prather, A.A., et al. (2016). Lifespan adversity and later adulthood telomere length in the nationally representative US Health and Retirement Study. *Proc Natl Acad Sci U S A* 113(42)**,** E6335-E6342. doi: 10.1073/pnas.1525602113.
Revesz, D., Milaneschi, Y., Terpstra, E.M., and Penninx, B.W. (2016). Baseline biopsychosocial determinants of telomere length and 6-year attrition rate. *Psychoneuroendocrinology* 67**,** 153-162. doi: 10.1016/j.psyneuen.2016.02.007.
Riley, G., Perrin, M., Vaez-Azizi, L.M., Ruby, E., Goetz, R.R., Dracxler, R., et al. (2018). Telomere length and early trauma in schizophrenia. *Schizophr Res*. doi: 10.1016/j.schres.2018.02.059.
Robles, T.F., Carroll, J.E., Bai, S., Reynolds, B.M., Esquivel, S., and Repetti, R.L. (2016). Emotions and family interactions in childhood: Associations with leukocyte telomere length emotions, family interactions, and telomere length. *Psychoneuroendocrinology* 63**,** 343-350. doi: 10.1016/j.psyneuen.2015.10.018.

Savolainen, K., Eriksson, J.G., Kananen, L., Kajantie, E., Pesonen, A.K., Heinonen, K., et al. (2014). Associations between early life stress, self-reported traumatic experiences across the lifespan and leukocyte telomere length in elderly adults. *Biol Psychol* 97**,** 35-42. doi: 10.1016/j.biopsycho.2014.02.002.

Schaakxs, R., Wielaard, I., Verhoeven, J.E., Beekman, A.T., Penninx, B.W., and Comijs, H.C. (2016). Early and recent psychosocial stress and telomere length in older adults. *Int Psychogeriatr* 28(3)**,** 405-413. doi: 10.1017/S1041610215001155.

Shalev, I., Moffitt, T.E., Braithwaite, A.W., Danese, A., Fleming, N.I., Goldman-Mellor, S., et al. (2014). Internalizing disorders and leukocyte telomere erosion: a prospective study of depression, generalized anxiety disorder and post-traumatic stress disorder. *Mol Psychiatry* 19(11)**,** 1163-1170. doi: 10.1038/mp.2013.183.

Shalev, I., Moffitt, T.E., Sugden, K., Williams, B., Houts, R.M., Danese, A., et al. (2013). Exposure to violence during childhood is associated with telomere erosion from 5 to 10 years of age: a longitudinal study. *Molecular psychiatry* 18(5)**,** 576-581.

Surtees, P.G., Wainwright, N.W., Pooley, K.A., Luben, R.N., Khaw, K.T., Easton, D.F., et al. (2011). Life stress, emotional health, and mean telomere length in the European Prospective Investigation into Cancer (EPIC)-Norfolk population study. *J Gerontol A Biol Sci Med Sci* 66(11)**,** 1152-1162. doi: 10.1093/gerona/glr112.

Tyrka, A.R., Parade, S.H., Price, L.H., Kao, H.T., Porton, B., Philip, N.S., et al. (2016). Alterations of Mitochondrial DNA Copy Number and Telomere Length With Early Adversity and Psychopathology. *Biol Psychiatry* 79(2)**,** 78-86. doi: 10.1016/j.biopsych.2014.12.025.

Tyrka, A.R., Price, L.H., Kao, H.T., Porton, B., Marsella, S.A., and Carpenter, L.L. (2010). Childhood maltreatment and telomere shortening: preliminary support for an effect of early stress on cellular aging. *Biol Psychiatry* 67(6)**,** 531-534. doi: 10.1016/j.biopsych.2009.08.014.

van Ockenburg, S.L., Bos, E.H., de Jonge, P., van der Harst, P., Gans, R.O., and Rosmalen, J.G. (2015). Stressful life events and leukocyte telomere attrition in adulthood: a prospective population-based cohort study. *Psychol Med* 45(14)**,** 2975-2984. doi: 10.1017/S0033291715000914.

Verhoeven, J.E., van Oppen, P., Puterman, E., Elzinga, B., and Penninx, B.W. (2015). The Association of Early and Recent Psychosocial Life Stress With Leukocyte Telomere Length. *Psychosom Med* 77(8)**,** 882-891. doi: 10.1097/PSY.0000000000000226.

Vincent, J., Hovatta, I., Frissa, S., Goodwin, L., Hotopf, M., Hatch, S.L., et al. (2017). Assessing the contributions of childhood maltreatment subtypes and depression case-control status on telomere length reveals a specific role of physical neglect. *J Affect Disord* 213**,** 16-22. doi: 10.1016/j.jad.2017.01.031.

Zalli, A., Carvalho, L.A., Lin, J., Hamer, M., Erusalimsky, J.D., Blackburn, E.H., et al. (2014). Shorter telomeres with high telomerase activity are associated with raised allostatic load and impoverished psychosocial resources. *Proc Natl Acad Sci U S A* 111(12)**,** 4519-4524. doi: 10.1073/pnas.1322145111.
